# Influence of Storage Environments and Packaging Materials on the Bioactive Compounds in Citri Reticulatae Pericarpium

**DOI:** 10.1002/fsn3.70393

**Published:** 2025-06-07

**Authors:** Mengdie Peng, Jinji Deng, Jiepei Xu, Baizhong Chen, Xiaojing Deng, Yi Cai, Wen Liu, Guodong Zheng

**Affiliations:** ^1^ Guangzhou Municipal and Guangdong Provincial Key Laboratory of Molecular Target & Clinical Pharmacology, The NMPA and State Key Laboratory of Respiratory Disease, School of Pharmaceutical Sciences and the Affiliated Traditional Chinese Medicine Hospital Guangzhou Medical University Guangzhou China; ^2^ Guangdong Xinbaotang Biological Technology Co., Ltd. Jiangmen China

**Keywords:** DPPH, GC–MS, Guang Chenpi, HPLC‐PDA, process of aging, UV

## Abstract

Guang Chenpi (GCP), a geographically protected citrus peel aged ≥ 3 years, is renowned for its medicinal and culinary value. However, scientific guidelines for optimizing its storage conditions—critical for balancing bioactive compound preservation and maturation—remain lacking, hindering industrial standardization. This study systematically evaluated how temperature, humidity, and container materials regulate GCP's chemical evolution during 24‐month storage. UV spectrophotometry quantified total flavonoids, polymethoxyflavones, and phenolic acids. HPLC‐PDA tracked eight bioactive markers (e.g., hesperidin and ferulic acid), while GC–MS analyzed volatile oils. Antioxidant activity was assessed via DPPH assays. The results demonstrated that low temperature and low humidity conditions (4°C ± 2°C/35%–40%) hinder the aging process of GCP, while prolonged exposure to high temperature or high humidity (35°C ± 2°C/> 85%) accelerates the transformation and formation of ferulic acid by 7‐fold but significantly decreases key components such as total flavonoids (decline rate doubled vs. normal temperature) and Methyl 2‐(methylamino) benzoate (1.59% → 0.14%). Notably, GCP stored in polyethylene (PE)‐sealed bags exhibited smaller reductions in total flavonoids and hesperidin, whereas those stored in gunny bags showed a significant decline in methyl 2‐(methylamino) benzoate content (1.59% → 1.05%). In conclusion, our findings demonstrate that storage conditions (temperature, humidity, and container type) critically govern the chemical trajectory of GCP aging. To standardize GCP quality, avoiding low temperature and low humidity and long‐term high temperature and high humidity conditions, as well as guaranteeing oxygen permeability (PE bags for late‐stage storage), is imperative, which will offer actionable guidelines to standardize GCP quality without compromising its heritage value.

## Graphical abstract

Guang Chenpi (GCP), a dual‐use food and medicine, requires optimal storage to maintain quality. Studies using UV, HPLC‐PDA, and GC–MS showed low temperature and humidity hinder aging, while high levels accelerate compound transformation but reduce key components like hesperidin. PE bags preserve flavonoids better than gunny bags. Scientific regulation of storage conditions is crucial for standardized GCP quality.

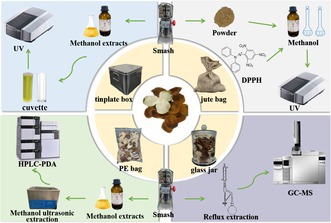

**Abbreviations**
5‐HPMF: 5‐hydroxy‐6,7,8,3′,4′‐pentamethoxyflavone
AEAC: ascorbic acid equivalent antioxidant capacity
CRP: Citri Reticulatae Pericarpium
DPPH: 2,2‐Diphenylpicrylhydrazyl
GC–MS: Gas chromatography–mass spectrometry
GCP: Guang Chenpi
HMF: 3,5,6,7,8,3′,4′‐heptamethoxyflavone
HPLC‐PDA: High performance liquid chromatography–Photo‐diode array
RSDs: relative standard deviations
TCM: traditional Chinese medicine
TFC: total flavonoid content
TLC: thin‐layer chromatography
TPC: total phenolic content
TPMFC: total polymethoxyflavone content
UV: ultraviolet–visible spectrophotometry

## 1 Introduction

*Citri Reticulatae Pericarpium* (CRP), commonly known as Chenpi in Chinese, refers to the dried mature peel of *Citrus reticulata Blanco* or its cultivated varieties (Pharmacopoeia CON 2020). The dried peel of *Citrus reticulata* ‘Chachi,’ referred to as Guang Chenpi (GCP), is a renowned medicinal resource from the Lingnan region (known as Daodi in Chinese), valued for its dual application in medicine and food. CRP is rich in diverse chemical constituents, including volatile components, flavonoids, alkaloids, and polysaccharides (Gao et al. 2023; Song et al. 2022; Zhang et al. 2024). As a traditional Chinese medicine (TCM) with medicinal and food homology, it exhibits significant pharmacological properties such as regulating qi, invigorating the spleen, alleviating vomiting, and reducing dampness and phlegm (Gao et al. 2023; Mei et al. 2019). Consequently, it is widely utilized in clinical applications as well as in the development and processing of non‐staple foods.

According to TCM theory, the pharmacological properties of CRP are enhanced after aging (Dai et al. 2023), aligning more closely with traditional therapeutic requirements. Typically, GCP is used after 3 years of natural aging, though it can be stored for up to 10 years or longer. However, standardization of the aging process remains a challenge. The local standard for GCP, Geographical Indication Products Xinhui Orange Peel DB 4407/T 70‐2021, specifies the “natural storage aging method in the protected area” as the official aging technique. Despite this, the quality of commercially available GCP varies widely due to the coexistence of naturally aged and technologically aged products. The latter involves accelerated aging under high temperature and high humidity conditions, leading to inconsistencies in quality.

Suitable storage conditions are critical to maintaining the quality of GCP (Yang, He, et al. 2022; Yang, Jiang, et al. 2022). Traditionally, GCP is aged in sacks, such as gunny bags, under ambient temperature and humidity. Storage containers, temperature, and humidity significantly influence the chemical transformations that occur during the aging process (Chen et al. 2024). Improper storage methods can result in issues such as staleness, insect infestation, or mildew, which severely impact the quality and pharmaceutical value of GCP.

Existing literature primarily focuses on the effects of bagged container storage on the quality of Tangerine peel, with limited studies addressing alternative storage containers like iron cabinets or glass jars. In practice, many enterprises store Tangerine peel in box‐type containers under controlled temperature conditions. According to Geographical Indication Product Xinhui Tangerine peel (DB 4407/T 70‐2021), the recommended storage temperature for Tangerine peel should not exceed 35°C. However, no research has systematically evaluated the quality impact of long‐term storage and aging at this temperature. The slow aging process at room temperature poses a significant challenge for scientific research. Accelerated aging experiments, utilizing chemical kinetic models such as the Arrhenius equation, have been shown to effectively simulate natural aging by reducing storage time under controlled conditions (Arcus and Mulholland 2020; Crapse et al. 2021).

This study focused on GCP as the primary research subject, investigating the effects of various storage environments and containers on the dynamic changes in its quality. The analysis encompassed volatile oils, flavonoids, organic acids, limonoids, and antioxidant activity, employing techniques such as UV–Vis spectroscopy, HPLC‐PDA, and GC–MS. The findings provide a scientific foundation for optimizing storage conditions during the industrialization of GCP and contribute to advancing the GCP industry.

## 2 Materials and Methods

### 2.1 Experiment Materials and Reagents

Approximately 100 kg of fresh, mature *Citrus reticulata* ‘Chachi’ fruits were collected from Shenlu Village, Sanjiang Town, Xinhui District, Jiangmen City, Guangdong Province, on December 20, 2019. After removing the pulp, the fresh peels were sun‐dried until their weight stabilized, following the standardized drying process for CRP. The moisture content of all CRP samples (6.5 kg) was below 13%. The samples were randomly divided into 12 groups, each weighing approximately 500 g. Four storage conditions were established to evaluate GCP stability under typical TCM storage scenarios: Group A (Standard conditions): 33°C–37°C, 65%–70% RH, following TCM storage guidelines. Group B (High temperature/Normal humidity): 33°C–37°C, 65%–70% RH, simulating tropical climates. Group C (High temperature/High humidity): 33°C–37°C, > 85% RH, replicating Xinhui monsoon‐like conditions. Group D (Low temperature/Low humidity): 2°C–6°C, 35%–40% RH, representing refrigerated storage. The remaining eight groups were stored in four different packaging materials (PE bags, jute bags, glass jars, and tinplate boxes) under high and normal temperature conditions. The PE bags (Guangzhou Bohe Biotechnology Co. Ltd., Guangdong Province) were made of polyethylene and measured 29 cm × 38 cm. The jute bags (Guangzhou Minghua Horticulture, Guangdong Province) were made of jute, measuring 35 cm × 50 cm, and were earthen yellow with moderate hardness and softness. The glass jars (Guangzhou Bohe Biotechnology Co. Ltd., Guangdong Province) were custom‐designed wide glass grinding bottles, with a diameter of 16 cm and a height of 21 cm. The tinplate boxes (Jiangmen Xinbaotang, Guangdong Province) were custom‐made, with a thickness of 0.35 mm, a capacity of 9 L, and dimensions of 23.5 cm × 23.5 cm × 17.5 cm. Table 1 provides detailed information on the grouping of GCP.

**TABLE 1:** Information of GCP samples in different storage methods.

Sample no.	Storage temperature	Storage humidity	Packaging materials
A	25°C ± 2°C	65%–70%	/
B	35°C ± 2°C	65%–70%	/
C	35°C ± 2°C	> 85%	/
D	4°C ± 2°C	35%–40%	/
A1	25°C ± 2°C	65%–70%	PE bag
A2	25°C ± 2°C	65%–70%	Jute bag
A3	25°C ± 2°C	65%–70%	Glass jar
A4	25°C ± 2°C	65%–70%	Tinplate box
B1	35°C ± 2°C	65%–70%	PE bag
B2	35°C ± 2°C	65%–70%	Jute bag
B3	35°C ± 2°C	65%–70%	Glass jar
B4	35°C ± 2°C	65%–70%	Tinplate box

Analytical‐grade methanol and ethyl acetate were obtained from Guanghua Technology Co. Ltd. (Guangzhou, China). HPLC‐grade methanol and n‐hexane were provided by Merck KGaA (Darmstadt, Germany), and acetonitrile was supplied by Honeywell International Inc. (New Jersey, USA). The 2,2‐diphenylpicrylhydrazyl (DPPH) reagent, purchased from Macklin Biochemical Co. Ltd. (Shanghai, China), was used to assess antioxidant activity.

The following standards were used for HPLC quantification: synephrine (99.53%), hesperidin (98.46%), ferulic acid (99.32%), limonin (98.30%), and tangeretin (99.77%), all sourced from Must Bio‐Technology Co. Ltd. (Sichuan, China). Nobiletin (98.86%) and 3,5,6,7,8,3′,4′‐heptamethoxyflavone (> 98.00%, HMF) were purchased from Weikeqi Biological Technology Co. Ltd. (Sichuan, China). Additionally, 5‐hydroxy‐6,7,8,3′,4′‐pentamethoxyflavone (> 98.00%, 5‐HPMF) was obtained from Spring & Autumn Biological Engineering Co. Ltd. (Jiangsu, China), and Vitamin C (> 99.00%) was obtained from Yuanye Bio‐Technology Co. Ltd. (Shanghai, China).

### 2.2 Preparation of the Sample for UV–vis Analysis

According to the method described by Zheng et al. (2020), with minor modifications, samples were prepared as follows: 0.2 g of GCP powder was extracted with 20 mL of methanol using an ultrasonic device (KQ‐800KDE, Kunshan Ultrasonic Instruments Co. Ltd., Jiangsu, China) at 40 kHz for 30 min. The extract was then filtered to obtain the GCP methanol extract for subsequent HPLC‐PDA analysis, as well as for the quantification of total flavonoid content (TFC) and total phenolic content (TPC). Similarly, 0.2 g of GCP powder was extracted twice with 20 mL of ethyl acetate, and the resulting filtrate constituted the GCP ethyl acetate extract used for determining the total polymethoxyflavone content (TPMFC). Standards of hesperidin, ferulic acid, nobiletin, and vitamin C were employed for quantification of TFC (283 nm), TPC (765 nm), TPMFC (330 nm), and antioxidant activity (517 nm), respectively.

### 2.3 Preparation of the Sample for HPLC‐PDA Quantification Analysis

The GCP methanol extract, as described in Section 2.2 (Zheng et al. 2020), was filtered through a 0.22 μm nylon membrane and analyzed using HPLC‐PDA (LC‐20AT, Shimadzu Co. Ltd., Kyoto, Japan) for quantification. Separation was performed on a Diamonsil C18 reversed‐phase column (4.6 mm × 250 mm, 5.0 μm, Beijing, China) at a flow rate of 0.7 mL·min^−1^ and a temperature of 30°C. Gradient elution was conducted using pH 3.70 phosphoric acid water (A) and a methanol‐acetonitrile mixture (2:1, B) under the following conditions: 0–5 min, 5% B; 5–10 min, 5%–60% B; 10–20 min, 60% B; 20–25 min, 60%–75% B; 25–30 min, 75%–80% B; 30–35 min, 80%–85% B; and 35–40 min, 85%–95% B. Quantification of eight phytochemicals was performed at specific wavelengths: 224 nm (synephrine), 209 nm (limonin), 283 nm (hesperidin), and 330 nm (nobiletin, HMF, tangeretin, and 5‐HPMF).

### 2.4 Preparation of the Sample for GC–MS Analysis

There are many methods for extracting volatile oils from GCP, and this study employs the steam distillation method (Pharmacopoeia CON 2020). Approximately 30 g of GCP samples were soaked in 300 mL of distilled water for 12 h in a round‐bottom flask. Volatile oil was then extracted from the GCP samples via steam distillation for 5 h. The determination method of volatile oil in CRP followed the Pharmacopoeia of China (2020, Volume I). The volatile oil content was calculated using the following formula: volatile oil content (g·kg^−1^) = volatile oil weight (g)/GCP sample weight (kg). For subsequent GC–MS analysis, 50 μL of volatile oil was diluted with 950 μL of *n*‐hexane and filtered through a 0.22 μm nylon membrane.

GC–MS analysis of the volatile oil was performed on a DB‐5MS column (30 m × 0.25 mm × 0.25 μm; Palo Alto, USA) using an Agilent gas chromatograph (7890A) coupled with a mass spectrometer (5975C; Palo Alto, USA). High‐purity helium (99%) served as the carrier gas at a flow rate of 1 mL·min^−1^, with a split ratio of 10:1. The injector and detector temperatures were set at 270°C. The temperature program was as follows: 60°C–80°C at 1°C·min^−1^, held at 80°C for 10 min; 80°C–250°C at 5°C·min^−1^; 250°C to 300°C at 20°C·min^−1^, and held at 300°C for 1 min. In EI+ mode, volatile components were ionized at 70 eV and scanned from *m/z* 30 to 550 amu at a speed of 0.2 scans·s^−1^. The identification of volatile components was performed by comparison with the NIST08.L library.

### 2.5 Preparation of the Sample for Evaluation of Antioxidant Activity

The antioxidant activity of GCP was assessed and calculated following the methods described in the references (Singh et al. 2021; Yu et al. 2022). To evaluate the antioxidant activity, 0.0037 g of DPPH reagent was accurately weighed and dissolved in methanol in a 100 mL volumetric flask to prepare the DPPH radical solution. Sample filtrates of 0.05, 0.10, 0.15, 0.20, 0.25, 0.30, 0.35, and 0.40 mL were collected and diluted to 1 mL with distilled water. Each diluted sample was mixed with 3 mL of DPPH radical solution, shaken thoroughly, and left in the dark for 30 min. The absorbance was measured at 517 nm using a UV spectrophotometer, with three replicates for each sample.

As a blank control, 1 mL of methanol was mixed with 3 mL of DPPH radical solution, shaken, and left in the dark for 30 min before measuring the absorbance at 517 nm. For the positive control, vitamin C was subjected to the same procedure. The percentage of DPPH radical scavenging was calculated, and a plot was constructed with mass concentration as the *x*‐axis and DPPH scavenging rate as the *y*‐axis. The IC50 value (the concentration at which 50% of the DPPH radicals are scavenged) was determined. The calculation formula is as follows:

$$\text{Radical}\;\text{scavenging}\;\text{rate}=1-\frac{\text{The absorbance of the test group}}{\text{The absorbance of the blank group}}$$

## 3 Results and Discussion

### 3.1 The Appearance Changes of GCP

**FIGURE 1:**
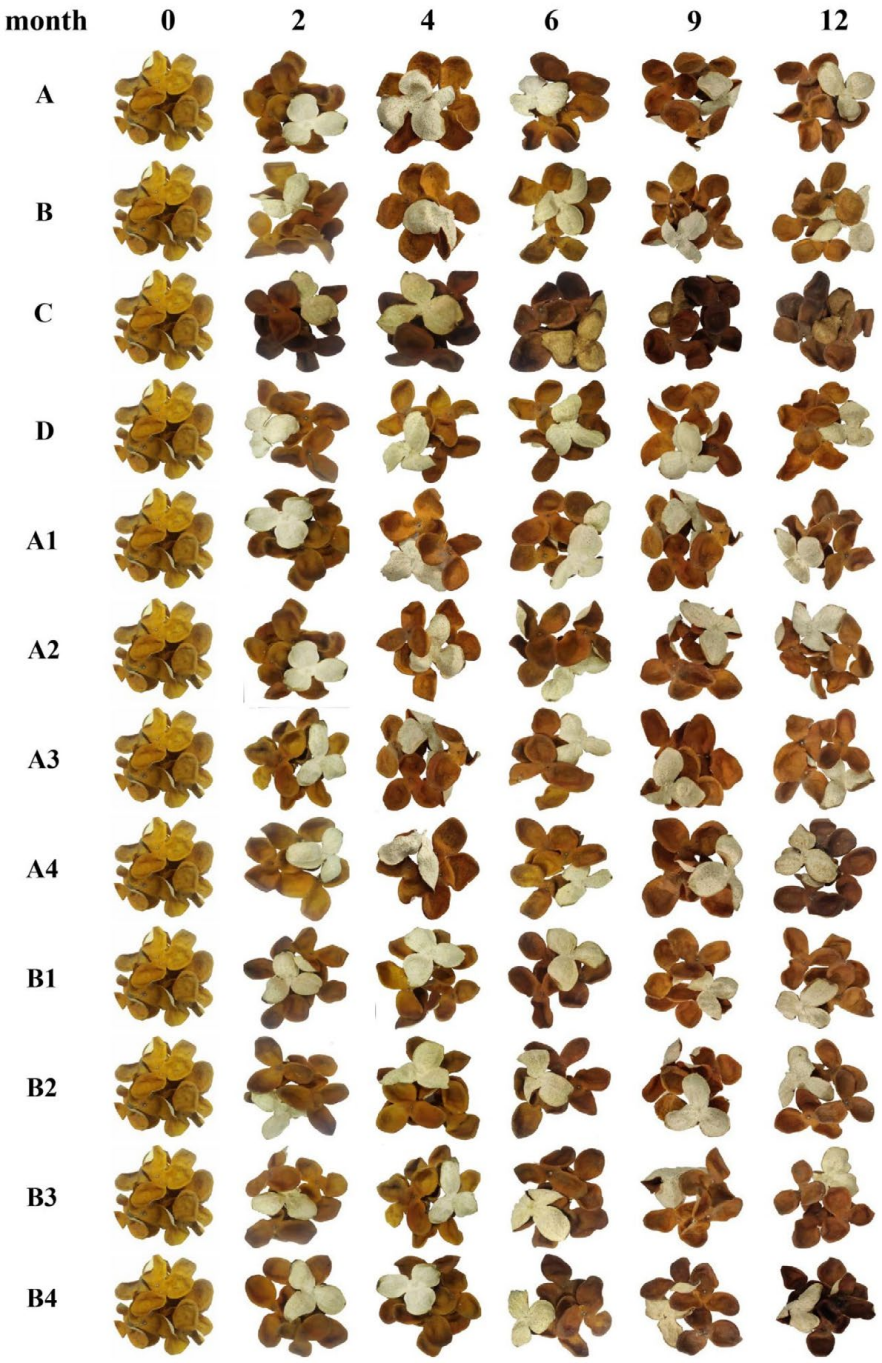
The appearance changes of the medicinal materials.

Under various storage conditions, the intact shape of the three petals connected at the base was maintained after storage periods of 0, 2, 4, 6, 9, and 12 months. The appearance changes of the medicinal materials are shown in Figure 1. The results indicated that the outer epidermis of GCP was light yellow at 0 months, with the color deepening as storage duration increased. Among the different storage environments, the order of color deepening, from most to least, was as follows: C (high temperature and high humidity), A (normal temperature and humidity), B (high temperature and normal humidity), and D (low temperature and low humidity). In group C, the color shifted to sepia, while in group A, it turned brownish‐yellow. For samples stored in different containers and temperatures, the color of Tangerine peel stored in tinplate boxes (A4 and B4) was darker compared to those stored in PE bags at both room temperature and high temperature. Additionally, the color of B4 was darker than that of A4.

Additionally, the color of the inner epidermis of group C (high temperature and high humidity) gradually deepened from light yellow‐white to sepia over time. The texture was soft and slightly moist, with a lighter aroma compared to the other groups and a slight occurrence of insect damage. In contrast, the color of the inner epidermis in groups A, B, and D showed no significant changes. The texture of group A was soft and pliable, while that of groups B and D was slightly hard and brittle. The yellow‐white tendon vascular bundle of the inner epidermis in group B had also significantly detached. Furthermore, under different storage temperatures, the texture of samples in groups A1–A4 remained soft and pliable, while those in groups B1–B4 were slightly hard and brittle. The fibers of the inner epidermis in the jute bags (A2 and B2) were more prone to shedding than in other groups.

### 3.2 Determination of TFC, TPMFC and TPC in Different Storage Methods

**FIGURE 2:**
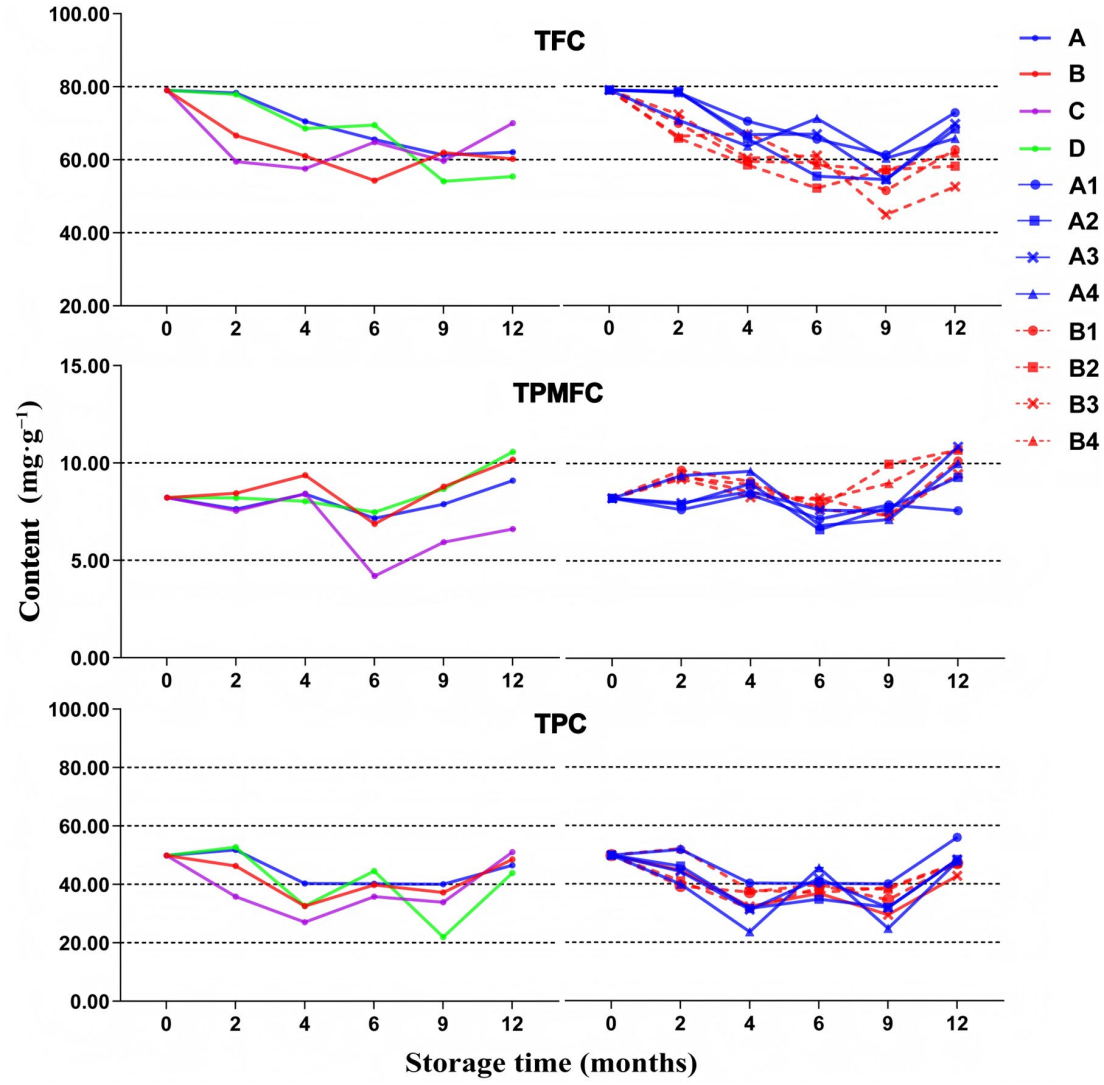
Line chart of total flavonoids, total polymethoxyflavones, and total phenolic acids content of GCP.

This study utilized the UV–Vis method to evaluate the dynamic changes in TFC, TPMFC, and TPC in GCP samples subjected to various environmental conditions and packaging materials over the course of 1 year. The content determination results are presented in Table 2 and Figure 2. SPSS 25.0 was used to analyze the changes in the contents of these compounds across four different storage environments over a period of 0–12 months. The trend analysis using the *χ* ^2^ test revealed a *χ* ^2^ value of 8.320, with no significant linear correlation (*p* > 0.05, *p* = 0.757). These results indicate no significant difference in the trends of total content for each component in relation to storage time across the different environments. Specifically, over 1 year, the TFC content in GCP decreased in all storage conditions. In contrast, the content of TPMFC initially decreased, then increased during the same period. Additionally, the TPC content exhibited greater variability across the different storage environments.

**TABLE 2:** Total flavonoids, polymethoxyflavones, and phenolic acid content of GCP in different storage environments (*n* = 3).

No	Storage environment	Storage time (month)	Content (mg·g^−1^)*		
			TFC	TPMFC	TPC
A	Normal temperature and humidity	0	79.002 ± 0.065	8.224 ± 0.000	49.886 ± 0.000
		2	78.302 ± 0.065	7.630 ± 0.000	51.827 ± 0.042
		4	70.478 ± 0.065	8.409 ± 0.000	40.302 ± 0.000
		6	65.565 ± 0.000	7.153 ± 0.010	40.198 ± 0.094
		9	61.330 ± 0.000	7.878 ± 0.000	40.009 ± 0.000
		12	62.096 ± 0.069	9.094 ± 0.000	46.547 ± 0.000
B	High temperature and normal humidity	0	79.002 ± 0.065	8.224 ± 0.000	49.886 ± 0.000
		2	66.632 ± 0.000	8.445 ± 0.000	46.328 ± 0.042
		4	61.019 ± 0.065	9.369 ± 0.000	32.521 ± 0.000
		6	54.289 ± 0.000	6.863 ± 0.010	39.798 ± 0.055
		9	61.996 ± 0.121	8.786 ± 0.000	37.176 ± 0.000
		12	60.234 ± 0.060	10.169 ± 0.010	48.515 ± 0.000
C	High temperature and high humidity	0	79.002 ± 0.065	8.224 ± 0.000	49.886 ± 0.000
		2	59.473 ± 0.000	7.539 ± 0.000	35.785 ± 0.000
		4	57.542 ± 0.065	8.415 ± 0.010	27.046 ± 0.000
		6	64.805 ± 0.000	4.195 ± 0.010	35.775 ± 0.055
		9	59.691 ± 0.069	5.926 ± 0.010	33.566 ± 0.054
		12	70.011 ± 0.000	6.603 ± 0.010	51.045 ± 0.000
D	Low temperature and low humidity	0	79.002 ± 0.065	8.224 ± 0.000	49.886 ± 0.000
		2	77.930 ± 0.000	8.210 ± 0.000	52.740 ± 0.042
		4	68.534 ± 0.065	8.028 ± 0.000	32.617 ± 0.083
		6	69.493 ± 0.000	7.473 ± 0.010	44.568 ± 0.000
		9	54.094 ± 0.069	8.669 ± 0.000	21.810 ± 0.000
		12	55.460 ± 0.000	10.565 ± 0.010	43.889 ± 0.000
A1	Normal temperature PE bag	0	79.002 ± 0.065	8.224 ± 0.000	49.886 ± 0.000
		2	78.302 ± 0.065	7.630 ± 0.000	51.827 ± 0.042
		4	70.478 ± 0.065	8.409 ± 0.000	40.302 ± 0.000
		6	65.565 ± 0.000	7.153 ± 0.010	40.198 ± 0.094
		9	61.330 ± 0.000	7.878 ± 0.000	40.009 ± 0.000
		12	72.851 ± 0.000	7.586 ± 0.000	55.919 ± 0.000
A2	Normal temperature jute bag	0	79.002 ± 0.065	8.224 ± 0.000	49.886 ± 0.000
		2	78.713 ± 0.000	7.902 ± 0.000	46.087 ± 0.000
		4	65.693 ± 0.000	8.964 ± 0.010	31.628 ± 0.104
		6	55.429 ± 0.000	6.604 ± 0.010	34.790 ± 0.000
		9	54.494 ± 0.000	7.760 ± 0.010	31.994 ± 0.000
		12	68.337 ± 0.000	9.295 ± 0.010	48.379 ± 0.000
A3	Normal temperature glass jar	0	79.002 ± 0.065	8.224 ± 0.000	49.886 ± 0.000
		2	78.377 ± 0.000	7.980 ± 0.010	44.286 ± 0.000
		4	66.814 ± 0.000	8.572 ± 0.000	31.225 ± 0.000
		6	66.959 ± 0.000	7.612 ± 0.000	41.864 ± 0.054
		9	54.514 ± 0.070	7.570 ± 0.000	31.838 ± 0.000
		12	69.763 ± 0.000	10.870 ± 0.000	48.345 ± 0.060
A4	Normal temperature tinplate box	0	79.002 ± 0.065	8.224 ± 0.000	49.886 ± 0.000
		2	70.771 ± 0.000	9.387 ± 0.000	39.940 ± 0.042
		4	63.674 ± 0.112	9.604 ± 0.000	23.588 ± 0.000
		6	71.266 ± 0.000	6.815 ± 0.000	45.541 ± 0.054
		9	60.349 ± 0.070	7.126 ± 0.000	24.762 ± 0.000
		12	65.778 ± 0.000	10.004 ± 0.000	47.705 ± 0.000
B1	High temperature PE bag	0	79.002 ± 0.065	8.224 ± 0.000	49.886 ± 0.000
		2	69.875 ± 0.000	9.658 ± 0.000	39.315 ± 0.000
		4	59.524 ± 0.112	9.085 ± 0.000	37.180 ± 0.083
		6	59.103 ± 0.000	7.642 ± 0.010	39.893 ± 0.055
		9	51.526 ± 0.000	7.343 ± 0.000	34.505 ± 0.000
		12	62.703 ± 0.000	10.127 ± 0.000	47.154 ± 0.000
B2	High temperature jute bag	0	79.002 ± 0.065	8.224 ± 0.000	49.886 ± 0.000
		2	65.849 ± 0.000	9.296 ± 0.000	41.044 ± 0.000
		4	58.477 ± 0.065	8.916 ± 0.000	31.657 ± 0.000
		6	52.177 ± 0.073	7.763 ± 0.010	38.692 ± 0.000
		9	57.212 ± 0.069	9.966 ± 0.010	38.218 ± 0.000
		12	58.163 ± 0.000	10.699 ± 0.010	47.049 ± 0.000
B3	High temperature glass jar	0	79.002 ± 0.065	8.224 ± 0.000	49.886 ± 0.000
		2	72.337 ± 0.000	9.206 ± 0.000	44.719 ± 0.000
		4	60.384 ± 0.065	8.264 ± 0.000	32.281 ± 0.083
		6	61.130 ± 0.000	8.234 ± 0.010	36.778 ± 0.054
		9	44.917 ± 0.000	7.317 ± 0.010	29.491 ± 0.000
		12	52.547 ± 0.000	9.467 ± 0.000	42.755 ± 0.000
B4	High temperaturetinplate box	0	79.002 ± 0.065	8.224 ± 0.000	49.886 ± 0.000
		2	66.334 ± 0.065	9.405 ± 0.000	52.139 ± 0.000
		4	67.188 ± 0.065	8.517 ± 0.000	37.853 ± 0.000
		6	58.470 ± 0.000	8.131 ± 0.010	37.060 ± 0.054
		9	57.129 ± 0.069	8.976 ± 0.017	38.780 ± 0.000
		12	61.736 ± 0.069	10.782 ± 0.000	47.179 ± 0.000

* Mean ± SD; *n* = 3.

In our investigation of the TFC content in broad citrus peel under varying environmental conditions, we found that, during the first 6 months of storage, the effects of low temperature and low humidity were similar to those of normal temperature and humidity conditions. However, after 9 months, the flavonoid content in the low temperature and low humidity group decreased at a rate three times faster than that in the normal conditions group. Interestingly, both groups exhibited a slight increase in TFC content after 12 months of storage. Nevertheless, the TFC content in the low temperature and low humidity group remained consistently lower than that in the normal temperature and humidity group. These findings suggest that the aging process of GCP requires specific temperature and humidity conditions. While low temperature and low humidity may preserve the freshness of GCP for a limited period, they are not conducive to the preservation of active substances during the aging process.

The decline in TFC content during the first 4 months was twice as rapid in the high temperature, normal humidity group and the high temperature, high humidity group compared to the normal temperature, normal humidity group and the low temperature, low humidity group. Notably, the turning point for increased flavonoid content occurred earlier in the high temperature, high humidity group (at 4 months) than in the high temperature, normal humidity group (at 6 months). These findings suggest that high temperature and humidity conditions can accelerate the aging process of Tangerine peel, possibly due to enhanced microbial activity. Previous studies have observed a decline in flavonoid content during the first year of storage at room temperature and humidity, followed by no significant changes or a slight increase after 1 year. However, because earlier studies only examined three time points—0, 1, and 2 years of storage—it was not possible to pinpoint the timing of the content increase. In this study, six time points were analyzed over 1 year, revealing that the turning point for flavonoid content increase under normal temperature and humidity conditions occurred at 9 months. Furthermore, prior research indicates that the number of fungal species on the surface of Tangerine peel peaks after 1 year of aging, with a subsequent reduction in both the diversity and total number of microbial species, ultimately leading to a more uniform composition (Yang et al. 2021).

This study examined the effects of various storage conditions on the concentrations of TFC, TPMFC, and TPC in GCP. The initial TFC content was 79.002 mg·g^−1^. Over 1 year of storage in different containers and under varying temperatures, a general decline in total flavonoid content was observed. At room temperature, the reduction ranged from 8% to 16%, while elevated temperatures caused a more significant decrease of 20%–33%, approximately twice the rate observed at room temperature. Notably, the reduction in flavonoid content was minimal in Tangerine peel stored in PE sealed bags, regardless of temperature conditions. Changes in TPC content across the eight samples exhibited similar patterns, with no significant differences detected after 12 months of storage. However, with the exception of sample A1 (normal temperature, PE bag), the TPMFC content in the other samples increased after 1 year, rising by 13%–32% compared to baseline levels.

### 3.3 Evaluation of Antioxidant Activity in Different Storage Methods

**FIGURE 3:**
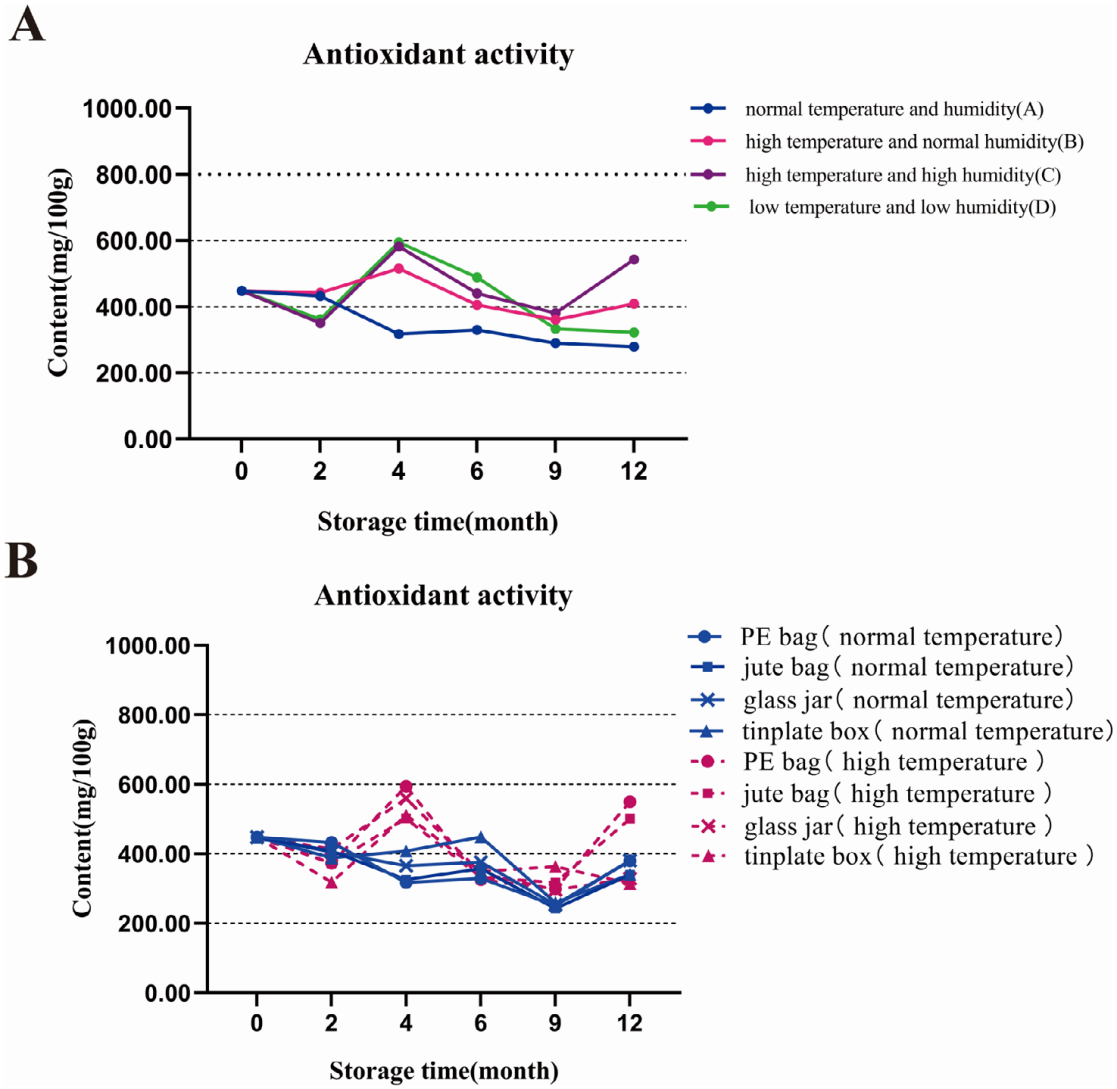
Comparison of antioxidant activity about GCP extracts in different storage environments.

The DPPH free radical scavenging assay is a widely used method for assessing the in vitro antioxidant activity of medicinal materials. In this study, the ascorbic acid equivalent antioxidant capacity (AEAC) was employed to evaluate the antioxidant capacity of Tangerine peel samples stored under different environmental conditions, with the results illustrated in Figure 3. After 12 months of storage, the antioxidant activity of the extracts decreased under normal temperature and humidity as well as under low temperature and humidity. Conversely, no significant change was observed under high temperature and normal humidity, while an increase in antioxidant activity was recorded under high temperature and high humidity, potentially due to the catalytic activity of antioxidant enzymes at 35°C. Furthermore, statistical correlation analysis revealed no absolute correlation between the content of individual components and antioxidant capacity. The antioxidant activity of medicinal materials is influenced by multiple compounds, including flavonoids, phenolic acids, polysaccharides, and others, suggesting that it is not solely dependent on any single component or class of components.

The results presented in Figure 3B demonstrate that after 12 months of storage at varying temperatures and in different containers, the antioxidant capacity of extracts stored at room temperature showed no significant differences across container types, although a slight decreasing trend was observed. Notably, the antioxidant capacity of Tangerine peel samples stored in PE sealed bags and jute bags increased at elevated temperatures. This enhancement may be attributed to the optimal high temperature of 35°C and the air permeability of these storage containers, which likely facilitated catalytic processes that improved the antioxidant capacity of the Tangerine peel.

### 3.4 Consecutive Changes of Eight Chemical Compositions in Different Storage Methods

**FIGURE 4:**
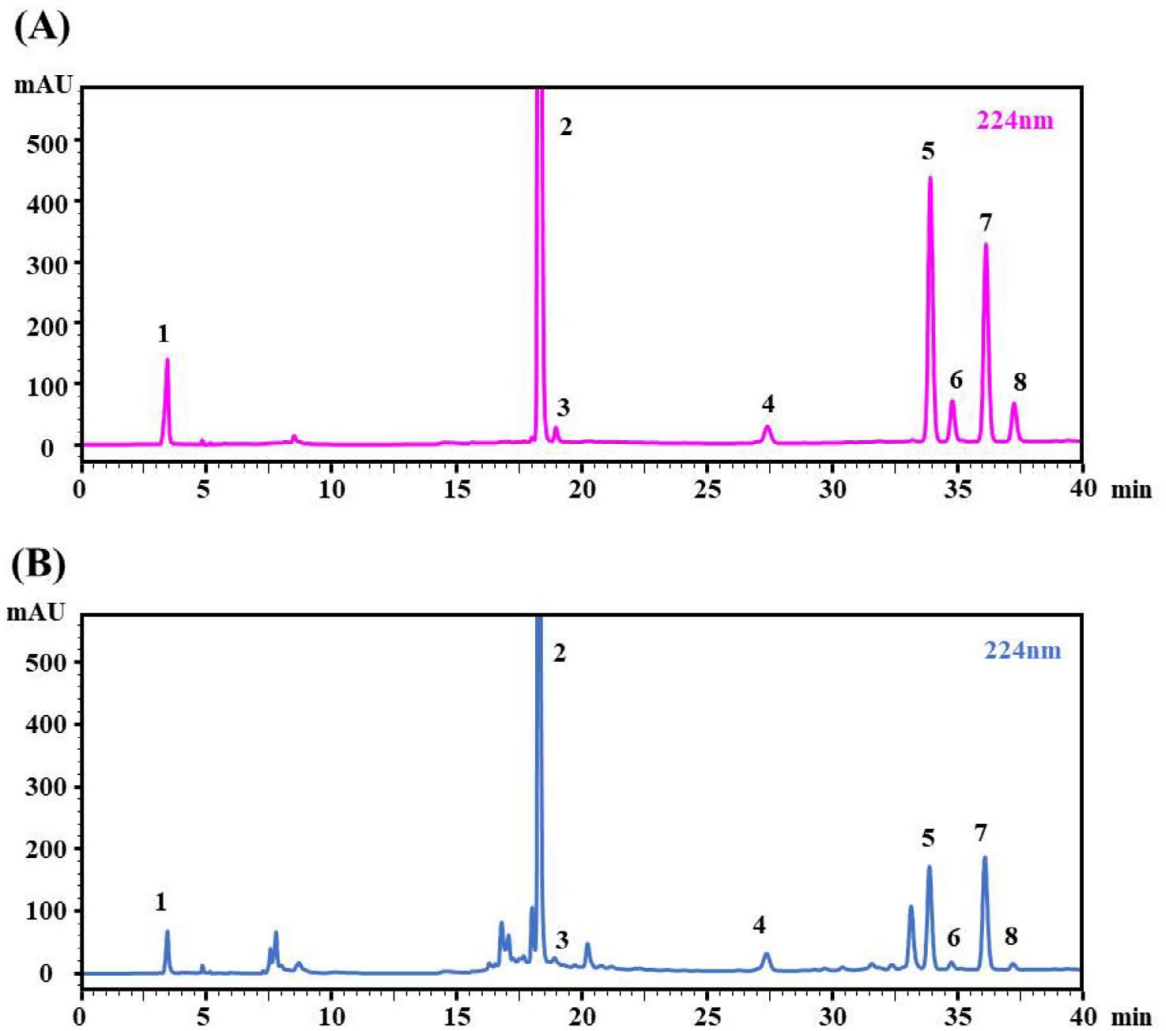
HPLC chromatogram of mixed reference standards (A) and *Citrus reticulata* ‘Chachi’ (B).

For the HPLC‐PDA quantitative analysis, eight chemical compositions were effectively separated within 40 min, with their peak areas used as indicators for methodological evaluation. The method demonstrated good linearity, stability, high precision, and repeatability, with relative standard deviations (RSDs) below 3%. The specific methodological results are shown in Table 3. The corresponding HPLC‐PDA chromatograms are presented in Figure 4.

**TABLE 3:** Validation of the HPLC‐PDA method.

Peak no.	Identification	Standard curve	*R* ^2^	Linearity range (μg·mL^−1^)	LOD (μg·mL^−1^)	LOQ (μg·mL^−1^)
1	Synephrine	*y* = 33,270*x* − 1833.1	0.9999	2.12–29.68	0.06375	0.08395
2	Hesperidin	*y* = 24,430*x* + 485,255	0.9990	77.06–539.42	0.01152	0.03840
3	Ferulic acid	*y* = 87,692*x* − 1245.6	0.9998	0.12–1.75	0.00961	0.03203
4	Limonin	*y* = 9804.3*x* + 19,996	0.9994	0.44–6.24	0.01834	0.02800
5	Nobiletin	*y* = 56,835*x* + 16,126	0.9999	5.04–70.56	0.00109	0.00365
6	HMF	*y* = 37,479*x*	0.9998	1.10–15.40	0.00824	0.02748
7	Tangeretin	*y* = 58,893*x*	0.9999	3.96–55.44	0.00534	0.01782
8	5‐HPMF	*y* = 37,816*x*	0.9999	1.12–15.68	0.00840	0.02801

**FIGURE 5:**
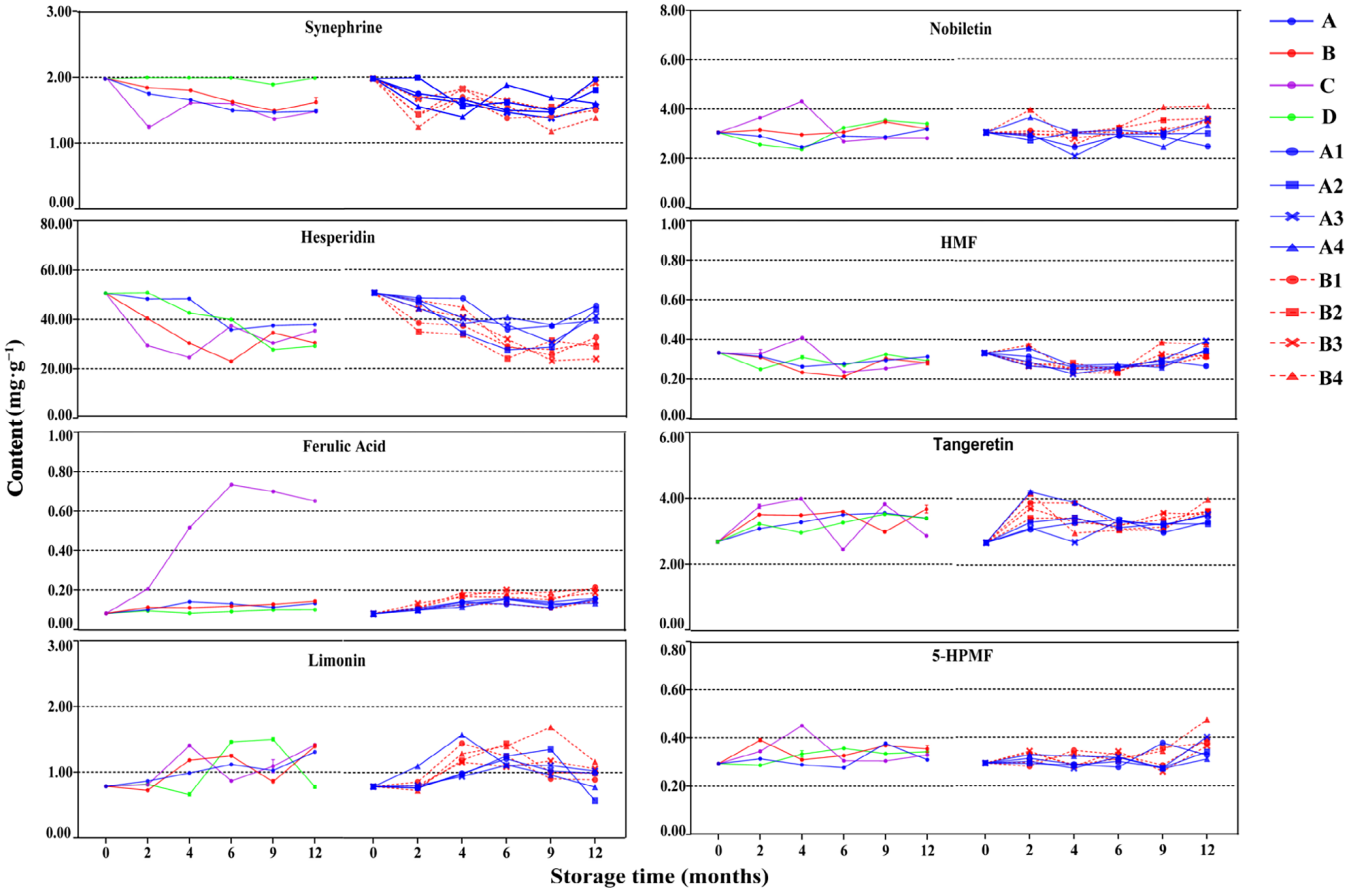
Line chart of 8 chemical compositions content of GCP in different storage environments.

The content determination results of the eight bioactive components are shown in Figure 5 and Table 4, revealing varying trends over a 12‐month storage period. Notably, hesperidin, the primary chemical component in GCP, is specified as a quality indicator in the Chinese Pharmacopeia (Pharmacopoeia CON 2020), with a minimum required content of 1.75%. HPLC‐PDA analysis showed a decreasing trend in hesperidin content across different storage environments and packaging materials. Among the four storage environments, the decline was slowest under normal temperature and humidity (Group A), with a 20% reduction over the study period. During the first 6 months, the decline rates in Groups A and D were nearly double those observed in Groups B and C, with Group D showing a pronounced decline after this period. Interestingly, an inflection point indicating a rise in hesperidin content was observed under certain conditions, with Group C showing this change after 4 months of storage. Regarding the four packaging materials, GCP samples stored in PE bags (Groups A1 and B1) exhibited the least decline in hesperidin content, regardless of storage at high or normal temperatures, compared to other containers.

**TABLE 4:** Eight chemical composition contents about GCP in different storage environments (*n* = 3).

No	Storage time (month)	Content (mg·g^−1^)*							
		Synephrine	Limonin	Hesperidin	Ferulic acid	Nobiletin	HMF	Tangeretin	5‐HPMF
A	0	1.976 ± 0.003	0.785 ± 0.004	50.563 ± 0.161	0.083 ± 0.000	3.049 ± 0.053	0.332 ± 0.004	2.679 ± 0.047	0.294 ± 0.003
	2	1.748 ± 0.024	0.866 ± 0.001	48.226 ± 0.336	0.101 ± 0.000	2.906 ± 0.007	0.314 ± 0.001	3.077 ± 0.016	0.314 ± 0.001
	4	1.657 ± 0.002	0.989 ± 0.033	48.320 ± 0.269	0.141 ± 0.001	2.466 ± 0.021	0.262 ± 0.006	3.270 ± 0.025	0.289 ± 0.001
	6	1.497 ± 0.004	1.118 ± 0.003	35.739 ± 0.140	0.131 ± 0.000	2.913 ± 0.012	0.277 ± 0.001	3.499 ± 0.002	0.277 ± 0.002
	9	1.468 ± 0.016	1.026 ± 0.002	37.408 ± 0.227	0.114 ± 0.002	2.871 ± 0.029	0.294 ± 0.001	3.549 ± 0.002	0.378 ± 0.003
	12	1.485 ± 0.024	1.308 ± 0.090	37.906 ± 0.285	0.134 ± 0.003	3.191 ± 0.032	0.312 ± 0.002	3.393 ± 0.025	0.310 ± 0.001
B	0	1.976 ± 0.003	0.785 ± 0.004	50.563 ± 0.161	0.083 ± 0.000	3.049 ± 0.053	0.332 ± 0.004	2.679 ± 0.047	0.294 ± 0.003
	2	1.840 ± 0.007	0.726 ± 0.010	40.427 ± 0.181	0.114 ± 0.001	3.151 ± 0.019	0.309 ± 0.003	3.498 ± 0.017	0.391 ± 0.006
	4	1.801 ± 0.000	1.184 ± 0.007	30.348 ± 0.087	0.111 ± 0.001	2.965 ± 0.007	0.233 ± 0.000	3.483 ± 0.011	0.310 ± 0.003
	6	1.625 ± 0.003	1.249 ± 0.023	22.992 ± 0.086	0.120 ± 0.000	3.061 ± 0.012	0.212 ± 0.004	3.592 ± 0.016	0.327 ± 0.002
	9	1.492 ± 0.007	0.857 ± 0.034	34.551 ± 0.275	0.129 ± 0.003	3.484 ± 0.021	0.303 ± 0.002	2.984 ± 0.026	0.369 ± 0.002
	12	1.620 ± 0.071	1.393 ± 0.035	30.498 ± 0.280	0.144 ± 0.005	3.202 ± 0.113	0.280 ± 0.010	3.668 ± 0.123	0.355 ± 0.014
C	0	1.976 ± 0.003	0.785 ± 0.004	50.563 ± 0.161	0.083 ± 0.000	3.049 ± 0.053	0.332 ± 0.004	2.679 ± 0.047	0.294 ± 0.003
	2	1.238 ± 0.035	0.811 ± 0.001	29.346 ± 0.341	0.207 ± 0.002	3.653 ± 0.044	0.327 ± 0.021	3.757 ± 0.064	0.345 ± 0.004
	4	1.612 ± 0.002	1.406 ± 0.012	24.591 ± 0.094	0.517 ± 0.001	4.310 ± 0.014	0.409 ± 0.001	3.989 ± 0.014	0.451 ± 0.001
	6	1.599 ± 0.005	0.868 ± 0.004	37.364 ± 0.004	0.734 ± 0.000	2.687 ± 0.001	0.235 ± 0.002	2.448 ± 0.036	0.306 ± 0.001
	9	1.365 ± 0.003	1.092 ± 0.099	30.440 ± 0.083	0.700 ± 0.003	2.823 ± 0.009	0.253 ± 0.004	3.820 ± 0.026	0.305 ± 0.002
	12	1.482 ± 0.003	1.416 ± 0.010	35.335 ± 0.137	0.652 ± 0.000	2.821 ± 0.009	0.284 ± 0.003	2.862 ± 0.008	0.330 ± 0.001
D	0	1.976 ± 0.003	0.785 ± 0.004	50.563 ± 0.161	0.083 ± 0.000	3.049 ± 0.053	0.332 ± 0.004	2.679 ± 0.047	0.294 ± 0.003
	2	1.997 ± 0.011	0.817 ± 0.021	50.818 ± 0.377	0.095 ± 0.001	2.573 ± 0.025	0.249 ± 0.003	3.223 ± 0.029	0.287 ± 0.002
	4	1.994 ± 0.004	0.663 ± 0.030	42.658 ± 0.145	0.084 ± 0.000	2.369 ± 0.004	0.309 ± 0.008	2.958 ± 0.001	0.334 ± 0.013
	6	1.990 ± 0.008	1.462 ± 0.017	39.937 ± 0.084	0.092 ± 0.001	3.240 ± 0.006	0.269 ± 0.002	3.262 ± 0.009	0.357 ± 0.002
	9	1.889 ± 0.014	1.499 ± 0.024	27.670 ± 0.068	0.100 ± 0.001	3.545 ± 0.008	0.324 ± 0.002	3.514 ± 0.010	0.335 ± 0.001
	12	1.987 ± 0.008	0.778 ± 0.022	29.14 5 ± 0.066	0.101 ± 0.000	3.409 ± 0.006	0.291 ± 0.004	3.392 ± 0.009	0.343 ± 0.000
A1	0	1.976 ± 0.003	0.785 ± 0.004	50.563 ± 0.161	0.083 ± 0.000	3.049 ± 0.053	0.332 ± 0.004	2.679 ± 0.047	0.294 ± 0.003
	2	1.748 ± 0.024	0.763 ± 0.001	48.431 ± 0.047	0.101 ± 0.000	2.906 ± 0.007	0.314 ± 0.001	3.077 ± 0.016	0.314 ± 0.001
	4	1.657 ± 0.002	0.989 ± 0.033	48.320 ± 0.269	0.141 ± 0.001	2.466 ± 0.021	0.262 ± 0.006	3.270 ± 0.025	0.289 ± 0.001
	6	1.497 ± 0.004	1.211 ± 0.000	35.739 ± 0.140	0.131 ± 0.000	2.913 ± 0.012	0.263 ± 0.001	3.371 ± 0.011	0.277 ± 0.002
	9	1.468 ± 0.016	1.026 ± 0.002	37.408 ± 0.227	0.114 ± 0.002	2.871 ± 0.029	0.294 ± 0.001	2.984 ± 0.026	0.378 ± 0.003
	12	1.963 ± 0.005	0.991 ± 0.043	45.373 ± 0.103	0.158 ± 0.000	2.480 ± 0.010	0.266 ± 0.006	3.305 ± 0.013	0.327 ± 0.003
A2	0	1.976 ± 0.003	0.785 ± 0.004	50.563 ± 0.161	0.083 ± 0.000	3.049 ± 0.053	0.332 ± 0.004	2.679 ± 0.047	0.294 ± 0.003
	2	1.988 ± 0.013	0.772 ± 0.058	46.736 ± 0.281	0.110 ± 0.000	2.728 ± 0.013	0.267 ± 0.002	3.290 ± 0.018	0.292 ± 0.005
	4	1.554 ± 0.009	0.972 ± 0.001	34.246 ± 0.045	0.143 ± 0.000	3.062 ± 0.002	0.249 ± 0.003	3.423 ± 0.006	0.286 ± 0.001
	6	1.616 ± 0.137	1.254 ± 0.003	27.518 ± 0.190	0.160 ± 0.000	3.140 ± 0.022	0.257 ± 0.001	3.129 ± 0.017	0.301 ± 0.001
	9	1.497 ± 0.003	1.357 ± 0.001	28.602 ± 0.079	0.141 ± 0.000	2.995 ± 0.002	0.275 ± 0.001	3.247 ± 0.000	0.274 ± 0.000
	12	1.802 ± 0.001	0.572 ± 0.043	43.786 ± 0.260	0.162 ± 0.000	3.007 ± 0.017	0.342 ± 0.003	3.241 ± 0.012	0.341 ± 0.000
A3	0	1.976 ± 0.003	0.785 ± 0.004	50.563 ± 0.161	0.083 ± 0.000	3.049 ± 0.053	0.332 ± 0.004	2.679 ± 0.047	0.294 ± 0.003
	2	1.698 ± 0.012	0.804 ± 0.106	47.578 ± 0.233	0.100 ± 0.001	2.976 ± 0.021	0.288 ± 0.004	3.116 ± 0.025	0.301 ± 0.003
	4	1.608 ± 0.005	0.941 ± 0.014	40.064 ± 0.057	0.130 ± 0.001	2.112 ± 0.005	0.226 ± 0.001	2.685 ± 0.003	0.272 ± 0.000
	6	1.465 ± 0.011	1.110 ± 0.011	37.666 ± 0.234	0.154 ± 0.001	2.961 ± 0.022	0.258 ± 0.000	3.345 ± 0.027	0.320 ± 0.003
	9	1.376 ± 0.007	1.109 ± 0.005	30.593 ± 0.135	0.125 ± 0.001	3.020 ± 0.013	0.297 ± 0.000	3.203 ± 0.018	0.274 ± 0.002
	12	1.550 ± 0.013	1.023 ± 0.029	41.101 ± 0.262	0.149 ± 0.001	3.592 ± 0.028	0.394 ± 0.008	3.520 ± 0.026	0.402 ± 0.005
A4	0	1.976 ± 0.003	0.785 ± 0.004	50.563 ± 0.161	0.083 ± 0.000	3.049 ± 0.053	0.332 ± 0.004	2.679 ± 0.047	0.294 ± 0.003
	2	1.551 ± 0.006	1.099 ± 0.104	44.325 ± 0.290	0.100 ± 0.000	3.656 ± 0.026	0.356 ± 0.005	4.214 ± 0.029	0.329 ± 0.003
	4	1.396 ± 0.025	1.577 ± 0.036	37.780 ± 0.004	0.116 ± 0.002	3.014 ± 0.049	0.269 ± 0.004	3.888 ± 0.062	0.323 ± 0.005
	6	1.880 ± 0.089	1.118 ± 0.011	40.710 ± 0.331	0.156 ± 0.001	2.970 ± 0.028	0.275 ± 0.000	3.301 ± 0.032	0.317 ± 0.004
	9	1.689 ± 0.006	0.963 ± 0.006	37.610 ± 0.035	0.133 ± 0.000	2.478 ± 0.000	0.259 ± 0.003	3.225 ± 0.000	0.271 ± 0.000
	12	1.599 ± 0.002	0.779 ± 0.019	39.469 ± 0.063	0.135 ± 0.001	3.337 ± 0.002	0.350 ± 0.002	3.486 ± 0.001	0.311 ± 0.005
B1	0	1.976 ± 0.003	0.785 ± 0.004	50.563 ± 0.161	0.083 ± 0.000	3.049 ± 0.053	0.332 ± 0.004	2.679 ± 0.047	0.294 ± 0.003
	2	1.427 ± 0.006	0.857 ± 0.028	38.419 ± 0.157	0.111 ± 0.001	3.109 ± 0.016	0.283 ± 0.003	3.876 ± 0.023	0.280 ± 0.003
	4	1.695 ± 0.009	1.447 ± 0.029	37.217 ± 0.396	0.170 ± 0.002	3.060 ± 0.028	0.257 ± 0.002	3.874 ± 0.036	0.347 ± 0.003
	6	1.377 ± 0.018	1.252 ± 0.011	28.985 ± 0.392	0.167 ± 0.002	3.048 ± 0.044	0.261 ± 0.004	3.209 ± 0.043	0.330 ± 0.006
	9	1.402 ± 0.018	0.907 ± 0.008	25.453 ± 0.321	0.153 ± 0.002	2.951 ± 0.039	0.268 ± 0.003	3.376 ± 0.010	0.285 ± 0.005
	12	1.495 ± 0.005	0.892 ± 0.009	32.615 ± 0.240	0.216 ± 0.002	3.504 ± 0.024	0.313 ± 0.004	3.626 ± 0.028	0.392 ± 0.002
B2	0	1.976 ± 0.003	0.785 ± 0.004	50.563 ± 0.161	0.083 ± 0.000	3.049 ± 0.053	0.332 ± 0.004	2.679 ± 0.047	0.294 ± 0.003
	2	1.670 ± 0.021	0.768 ± 0.010	34.834 ± 0.157	0.107 ± 0.002	2.953 ± 0.015	0.271 ± 0.003	3.413 ± 0.023	0.297 ± 0.009
	4	1.819 ± 0.007	1.187 ± 0.026	33.692 ± 0.035	0.128 ± 0.001	3.026 ± 0.003	0.282 ± 0.003	3.415 ± 0.001	0.332 ± 0.001
	6	1.641 ± 0.010	1.443 ± 0.009	24.051 ± 0.110	0.133 ± 0.001	3.232 ± 0.019	0.239 ± 0.004	3.075 ± 0.017	0.306 ± 0.021
	9	1.506 ± 0.004	1.000 ± 0.023	31.358 ± 0.022	0.109 ± 0.007	3.546 ± 0.024	0.305 ± 0.017	3.145 ± 0.038	0.358 ± 0.011
	12	1.911 ± 0.005	0.991 ± 0.030	28.848 ± 0.001	0.146 ± 0.001	3.599 ± 0.004	0.318 ± 0.002	3.627 ± 0.005	0.378 ± 0.001
B3	0	1.976 ± 0.003	0.785 ± 0.004	50.563 ± 0.161	0.083 ± 0.000	3.049 ± 0.053	0.332 ± 0.004	2.679 ± 0.047	0.294 ± 0.003
	2	1.242 ± 0.004	0.803 ± 0.001	44.249 ± 0.013	0.134 ± 0.001	3.020 ± 0.005	0.266 ± 0.001	3.709 ± 0.002	0.344 ± 0.001
	4	1.702 ± 0.019	1.160 ± 0.026	40.755 ± 0.006	0.171 ± 0.001	2.828 ± 0.003	0.254 ± 0.000	3.290 ± 0.005	0.282 ± 0.002
	6	1.575 ± 0.005	1.092 ± 0.016	31.877 ± 0.041	0.204 ± 0.001	2.997 ± 0.004	0.236 ± 0.001	3.185 ± 0.005	0.342 ± 0.009
	9	1.175 ± 0.011	1.178 ± 0.015	23.152 ± 0.149	0.164 ± 0.001	3.150 ± 0.023	0.326 ± 0.005	3.566 ± 0.022	0.259 ± 0.001
	12	1.380 ± 0.011	1.056 ± 0.051	23.872 ± 0.143	0.188 ± 0.000	3.522 ± 0.006	0.318 ± 0.002	3.522 ± 0.003	0.366 ± 0.001
B4	0	1.976 ± 0.003	0.785 ± 0.004	50.563 ± 0.161	0.083 ± 0.000	3.049 ± 0.053	0.332 ± 0.004	2.679 ± 0.047	0.294 ± 0.003
	2	1.434 ± 0.011	0.729 ± 0.016	47.530 ± 0.440	0.114 ± 0.000	3.965 ± 0.006	0.372 ± 0.005	4.159 ± 0.007	0.337 ± 0.008
	4	1.821 ± 0.008	1.288 ± 0.010	44.479 ± 0.078	0.189 ± 0.001	2.568 ± 0.001	0.233 ± 0.001	2.968 ± 0.002	0.288 ± 0.004
	6	1.478 ± 0.016	1.406 ± 0.022	28.521 ± 0.161	0.183 ± 0.001	3.279 ± 0.017	0.231 ± 0.001	3.056 ± 0.012	0.312 ± 0.001
	9	1.545 ± 0.006	1.693 ± 0.083	27.604 ± 0.049	0.191 ± 0.000	4.072 ± 0.001	0.385 ± 0.004	3.057 ± 0.023	0.344 ± 0.001
	12	1.521 ± 0.015	1.165 ± 0.036	30.491 ± 0.005	0.210 ± 0.000	4.109 ± 0.001	0.376 ± 0.001	3.971 ± 0.001	0.474 ± 0.001

* Mean ± SD; *n* = 3.

In contrast to the observed changes in hesperidin, the content of ferulic acid showed an increasing trend across various storage environments and packaging materials. Modern pharmacological studies indicate that ferulic acid is widely used in the pharmaceutical, food, and cosmetics industries. Clinically, ferulic acid exhibits antioxidant (Salau et al. 2022; Yin et al. 2019), anti‐inflammatory (Shi et al. 2021), anti‐thrombosis (Guo et al. 2022; Xie et al. 2021), and antibacterial properties (Kang et al. 2020; Mishra et al. 2022). Moreover, ferulic acid and its derivatives have become important targets in the design and synthesis of anti‐Alzheimer's disease medications (Di Giacomo et al. 2022; Ermis et al. 2023; Phadke et al. 2021; Singh et al. 2020). In the food industry, it serves as an antioxidant, while in cosmetics, it functions as a sunscreen, antioxidant, and stabilizer. Previous research by our group revealed that ferulic acid content was initially low at the time of harvest but increased with extended storage duration. In the present study, we observed that the ferulic acid content of GCP samples rose significantly after 1 year of storage under various conditions, consistent with earlier findings for samples stored for different durations. Notably, under conditions of high temperature and high humidity (A), the ferulic acid content increased sevenfold compared to other environments. This substantial increase may be attributed to enzyme catalysis promoted by the optimal combination of elevated temperature and humidity.

The aging process of Tangerine peel is marked by a natural phenomenon in which prolonged storage results in a darker appearance. Previous studies have shown that compounds such as ferulic acid, 4‐hydroxybenzoic acid, and caffeic acid contribute to the browning of medicinal materials, with the type and concentration of phenolic components directly influencing the degree of browning (Tang et al. 2020; Zhang et al. 2021). Research analyzing phenolic acid components in GCP across different aging periods identified ferulic acid as the most abundant phenolic acid, with its concentration increasing significantly over a storage period of 0–3 years (Yang, Jiang, et al. 2022). Building on existing research (Chao et al. 2022) and our accelerated aging experiments, we found that ferulic acid consistently increased during the aging process of GCP. This observation suggests a potential application for using ferulic acid content as a basis for developing an aging‐year discrimination model for GCP. However, further studies are needed to clarify whether a correlation exists between the storage duration, appearance color, and ferulic acid content of GCP.

The results revealed that after 12 months of storage, the ferulic acid content in GCP increased more significantly at elevated temperatures compared to normal temperatures across all four container types. This observation suggests that higher temperatures may enhance the activity of feruloyl esterase in the crude medicine. Previous studies have shown that feruloyl esterase degrades ferulic acid in plant cells through the hydrolysis of the cross‐linked cellulose network within plant cell walls (Jeon et al. 2023). Feruloyl esterase is naturally derived not only from plant cells (Hassan and Hugouvieux‐Cotte‐Pattat 2011; Oliveira et al. 2020) but also from animals and microorganisms (Duan et al. 2021; Liu et al. 2021; Mafa et al. 2021). Among these sources, Aspergillus niger, a fungus known to secrete feruloyl esterase, is the predominant species identified in GCP (Wang et al. 2021). After 1 year of storage under varying temperatures and in different containers, the hesperidin content consistently showed a declining trend, which was more pronounced at elevated temperatures than at normal temperatures. Moreover, samples stored in polyethylene bags (A1 and B1) exhibited a relatively smaller decrease in hesperidin content under both temperature conditions. In contrast, the levels of tangeretin and 5‐hydroxy‐6,7,8,3′,4′‐pentamethoxyflavone increased under various storage conditions, diverging from the pattern observed for hesperidin.

### 3.5 Consecutive Changes of Volatile Constituents in Different Storage Methods

#### 3.5.1 Extraction Rate of Essential Oil of GCP Samples

The extraction rates of volatile oil from Tangerine peel stored in various environments and containers are shown in Table 5. One‐way analysis of variance (ANOVA), performed using SPSS 25.0, revealed no significant differences in the volatile oil content of Tangerine peel after 0, 2, 4, 6, 9, and 12 months of storage across the four environments (*p* > 0.05). Consequently, it cannot be concluded that the storage environments utilized in this study had differing effects on the preservation of GCP. Besides, SPSS 25.0 was used to analyze the volatile oil content of GCP under 8 different storage conditions at 0, 2, 4, 6, 9, and 12 months. Firstly, the Kruskal–Wallis H test was used to compare the differences of different storage conditions at each time point. The results showed that there was no significant difference in the content of essential oil between the eight groups of GCP samples under high temperature and room temperature (four different containers) storage conditions (*p* = 0.43–1.00 > 0.05). One‐way ANOVA was used to investigate the effect of storage conditions on the dynamic change of volatile oil content, and the results showed that different storage conditions had no significant effect on the changing trend of volatile oil content in GCP (*p* = 0.636 > 0.05).

**TABLE 5:** Extraction rate of essential oil of GCP samples in different storage methods.

No.	Storage environment	Storage humidity	Packaging materials	Extraction rate of essential oil (%)					
				0 month	2 months	4 months	6 months	9 months	12 months
A	23°C ± 2°C	65%–70%	/	3.43	3.86	3.9	4.4	2.92	3.32
B	35°C ± 2°C	65%–70%	/	3.43	4.53	6.03	4.97	3.64	3.23
C	35°C ± 2°C	> 85%	/	3.43	4	4.08	3.43	3.85	3.9
D	4°C ± 2°C	35%–40%	/	3.43	4.4	4.69	3.97	3.57	3.35
A1	25°C ± 2°C	65%–70%	PE bag	3.43	3.86	3.9	4.4	2.92	3.32
A2	25°C ± 2°C	65%–70%	Jute bag	3.43	4.53	6.03	4.97	3.64	3.23
A3	25°C ± 2°C	65%–70%	Glass jar	3.43	4	4.08	3.43	3.85	3.9
A4	25°C ± 2°C	65%–70%	Tinplate box	3.43	4.4	4.69	3.97	3.57	3.35
B1	35°C ± 2°C	65%–70%	PE bag	3.43	4.6	5.61	5.59	3.26	3.11
B2	35°C ± 2°C	65%–70%	Jute bag	3.43	4.35	5.35	5.79	3.97	3.34
B3	35°C ± 2°C	65%–70%	Glass jar	3.43	4.81	4.95	5.31	3.59	3.09
B4	35°C ± 2°C	65%–70%	Tinplate box	3.43	4.38	4.7	3.85	4.53	3.54

The volatile oil content is largely determined by the size and number of spot‐like oil chambers in the outer epidermis of the peel. Since a uniform batch of GCP samples was used, variations in storage environments and containers did not affect the physical properties of these oil chambers. As a result, the volatile oil content remained stable after 1 year of storage under the tested conditions, regardless of temperature or container type. Therefore, during aging and long‐term storage, it is essential to preserve the integrity of the herbal material's structure. This approach ensures better retention of volatile oil within the oil chambers and prevents loss due to structural damage.

#### 3.5.2 Volatile Component Identification Results in GCP

##### 3.5.2.1 Volatile Components in GCP Under Different Storage Conditions

**FIGURE 6:**
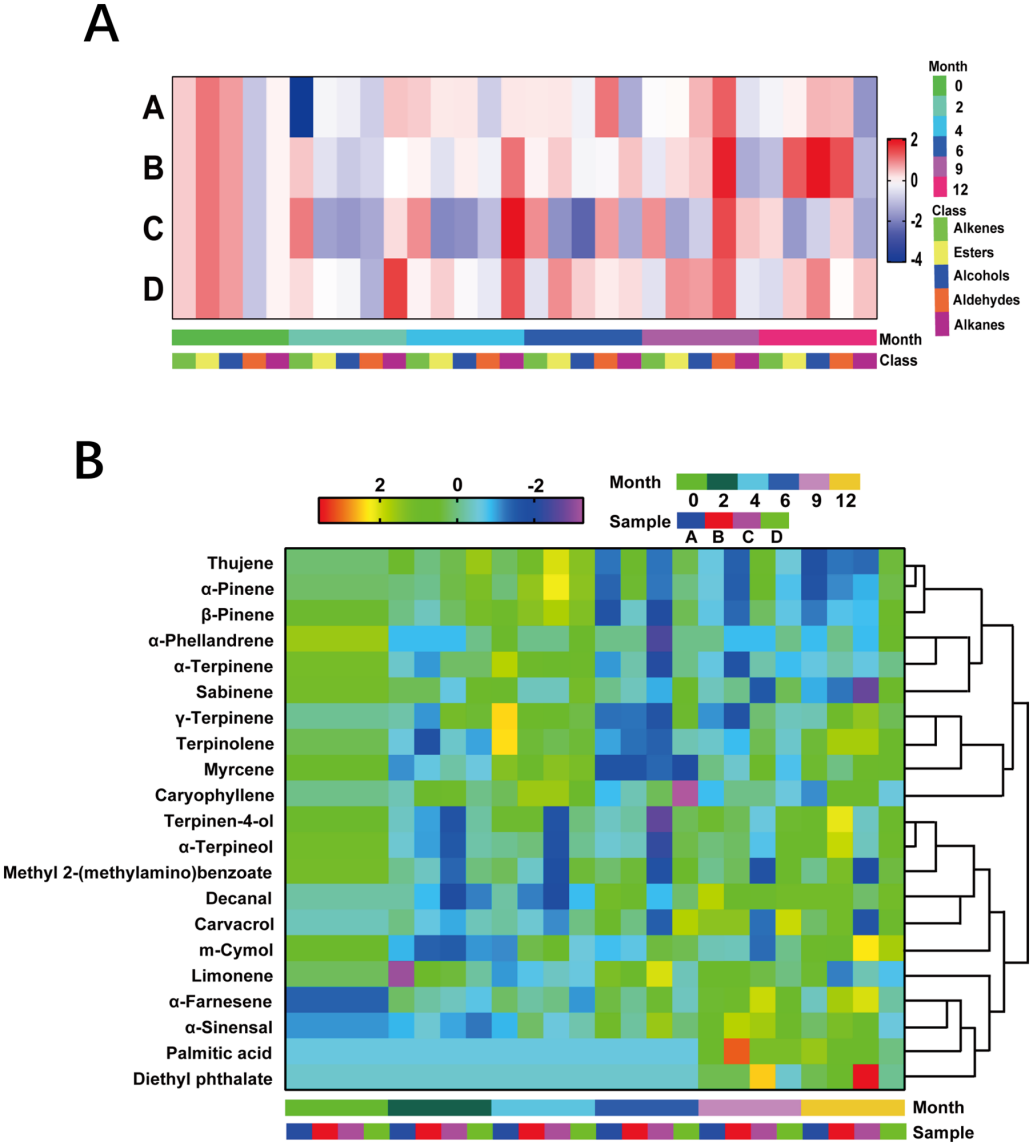
Heat map of relative contents of different kinds of volatile components of GCP in different storage environments.

The total values of various volatile components in citrus peel stored under four different environmental conditions are presented in Table 6. The thermogram of relative contents, normalized using SPSS 25.0, is shown in Figure 6. After 1 year of storage at normal temperature and humidity (A), high temperature and constant humidity (B), and high temperature and high humidity (C), the contents of alkenes and alkanes decreased. Notably, the relative contents of esters and alcohols significantly increased under high temperature and constant humidity (B), while these components decreased under high temperature and high humidity (C). Conversely, after 1 year of storage at low temperature and low humidity (D), the content of alkenes decreased, whereas other components remained largely unchanged. These findings indicate a general decreasing trend in the content of alkenes across different storage environments, while esters and alcohols exhibited varying patterns depending on the specific storage conditions.

**TABLE 6:** Relative contents of different kinds of volatile components of GCP in different storage environments.

NO.	Storage conditions	Storage time (month)	Relative contents of various volatile components (%)					
			Alkene	Ester	Alcohol	Aldehyde	Alkanes	Others
A	Normal temperature and humidity	0	96.27	1.59	0.66	0.29	0.62	1.54
		2	90.32	1.00	0.50	0.31	0.66	1.08
		4	96.27	1.15	0.57	0.31	0.63	1.00
		6	95.95	1.16	0.51	0.61	0.47	1.16
		9	96.65	1.09	0.63	0.64	0.56	1.43
		12	95.49	1.12	0.63	0.52	0.43	1.76
B	High temperature and normal humidity	0	96.27	1.59	0.66	0.29	0.62	1.54
		2	96.32	0.85	0.41	0.33	0.61	0.88
		4	95.84	0.84	0.56	0.40	0.73	1.43
		6	95.85	1.27	0.52	0.42	0.66	1.15
		9	95.24	1.20	0.61	0.73	0.47	1.50
		12	94.33	1.70	0.85	0.66	0.49	1.75
C	High temperature and high humidity	0	96.27	1.59	0.66	0.29	0.62	1.54
		2	97.14	0.36	0.29	0.22	0.64	0.83
		4	96.93	0.17	0.27	0.27	0.83	1.50
		6	96.93	0.24	0.17	0.56	0.47	1.38
		9	96.90	0.38	0.42	0.67	0.66	0.93
		12	96.14	0.30	0.43	0.50	0.46	2.05
D	Low temperature and low humidity	0	96.27	1.59	0.66	0.29	0.62	1.54
		2	96.10	1.05	0.52	0.24	0.78	1.01
		4	95.85	1.25	0.53	0.38	0.76	1.19
		6	95.10	1.50	0.60	0.46	0.64	1.56
		9	95.10	1.48	0.66	0.64	0.57	1.56
		12	94.96	1.26	0.69	0.44	0.66	1.96

**FIGURE 7:**
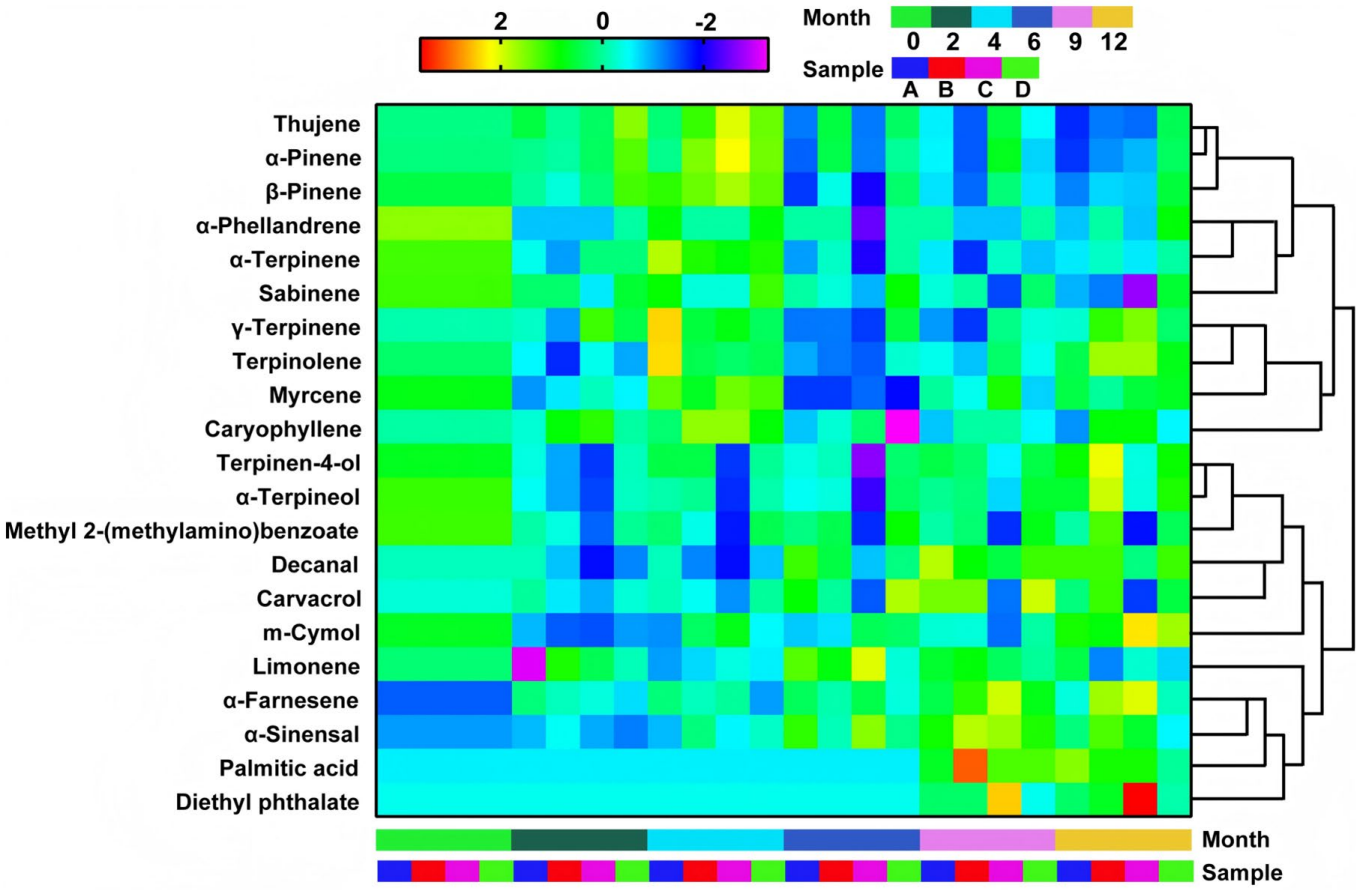
Heat map of relative contents of 21 volatile components of GCP in different storage environments.

A total of 21 major volatile components with a relative percentage greater than 0.10% were identified from GCP under various storage environments, including 12 alkenes, 2 esters, 2 alcohols, 2 aldehydes, and 3 other components. The relative contents of these components are presented in Table A1 in the Appendix A. Using SPSS 25.0, the relative contents were normalized, and thermal spectra of the major volatile components under different storage conditions were generated, as shown in Figure 7. The analysis revealed that the relatively high content of limonene in GCP remained stable after 1 year of storage at room temperature and humidity. However, its relative content decreased under conditions of high temperature and high humidity, high temperature and low humidity, and low temperature and humidity. Additionally, under high temperature and high humidity conditions, the relative contents of carvacrol and methyl 2‐(methylamino) benzoate significantly decreased, with the relative content of methyl 2‐(methylamino) benzoate dropping to 0.14%. Concurrently, diethyl phthalate exhibited a marked accumulation during storage (undetectable at 0/2/4/6 months), suggesting that methyl 2‐(methylamino) benzoate serves as a precursor in chemical transformation processes under these conditions. Critically, the sharp decline in methyl 2‐(methylamino) benzoate levels (↓64.5% ± 3.8%) resulted in the absence of characteristic fluorescent spots during thin‐layer chromatography (TLC) identification as mandated by the Chinese Pharmacopeia (2020 edition, Volume IV, Appendix VI B), thereby rendering the samples non‐compliant with pharmacopeial quality standards for medicinal materials (Content limit: ≥ 0.15% w/w).

##### 3.5.2.2 Volatile Components in GCP Under Different Storage Containers

Table 7 presents the total volatile component content in GCP samples stored at different temperatures and in various containers. The analysis shows that alkenes are the predominant volatile components in GCP, accounting for up to 90% of the total content. Esters, primarily represented by methyl 2‐(methylamino) benzoate, a characteristic component of GCP, make up more than 1% of the total volatile components.

**TABLE 7:** Relative contents of various volatile components of GCP in different packaging materials.

No.	Storage conditions	Storage time (month)	Relative contents of various volatile components (%)					
			Alkene	Ester	Alcohol	Aldehyde	Alkanes	Others
A1	Normal temperature PE bag	0	96.27	1.59	0.66	0.29	0.62	1.54
		2	91.25	1.00	0.5	0.31	0.66	1.08
		4	96.27	1.15	0.57	0.31	0.63	1.00
		6	95.95	1.16	0.51	0.61	0.47	1.16
		9	94.86	1.41	0.65	0.82	0.56	1.46
		12	93.92	1.83	0.64	0.62	0.47	2.43
A2	Normal temperature jute bag	0	96.27	1.59	0.66	0.29	0.62	1.54
		2	92.7	1.05	0.42	0.40	0.5	0.88
		4	96.83	0.82	0.44	0.26	0.77	0.85
		6	96.23	0.97	0.36	0.51	0.58	1.27
		9	95.87	1.18	0.63	0.65	0.65	1.53
		12	94.54	1.15	0.59	0.44	0.62	2.69
A3	Normal temperature glass jar	0	96.27	1.59	0.66	0.29	0.62	1.54
		2	93.59	1.28	0.62	0.28	0.66	0.97
		4	95.2	1.41	0.68	0.25	0.88	1.51
		6	95.12	1.67	0.74	0.61	0.51	1.26
		9	94.67	1.69	0.94	0.62	0.51	1.55
		12	92.74	2.17	1.09	0.68	0.53	2.46
A4	Normal temperature tinplate box	0	96.27	1.59	0.66	0.29	0.62	1.54
		2	94.42	1.23	0.65	0.33	0.53	0.92
		4	95.32	1.54	0.73	0.35	0.73	1.27
		6	94.73	1.60	0.68	0.46	0.69	1.76
		9	94.48	1.86	0.78	0.69	0.52	1.67
		12	93.32	2.08	1.08	0.55	0.59	2.23
B1	High temperature PE bag	0	96.27	1.59	0.66	0.29	0.62	1.54
		2	95.48	0.87	0.49	0.17	0.52	1.02
		4	96.24	0.72	0.45	0.31	0.73	1.46
		6	95.92	0.84	0.44	0.46	0.63	1.57
		9	95.2	1.39	0.58	0.74	0.55	1.41
		12	94.69	1.3	0.79	0.62	0.5	1.98
B2	High temperature jute bag	0	96.27	1.59	0.66	0.29	0.62	1.54
		2	95.63	0.78	0.54	0.14	0.69	1.22
		4	96.00	0.81	0.54	0.29	0.81	1.45
		6	96.13	1.05	0.49	0.52	0.49	1.11
		9	95.41	1.33	0.65	0.67	0.52	1.26
		12	94.29	1.59	0.81	0.62	0.48	1.97
B3	High temperature glass jar	0	96.27	1.59	0.66	0.29	0.62	1.54
		2	95.61	1.00	0.57	0.23	0.63	0.91
		4	95.19	1.42	0.7	0.35	0.79	1.52
		6	95.00	1.71	0.72	0.66	0.46	1.33
		9	94.82	1.61	0.82	0.61	0.51	1.63
		12	94.42	1.76	0.98	0.56	0.5	1.75
B4	High temperature tinplate box	0	96.27	1.59	0.66	0.29	0.62	1.54
		2	95.74	1.23	0.68	0.27	0.59	0.97
		4	95.07	1.36	0.73	0.31	0.73	1.72
		6	95.26	1.5	0.71	0.46	0.55	1.49
		9	94.91	1.45	0.86	0.51	0.63	1.64
		12	93.51	2.04	1.17	0.55	0.55	2.09

**FIGURE 8:**
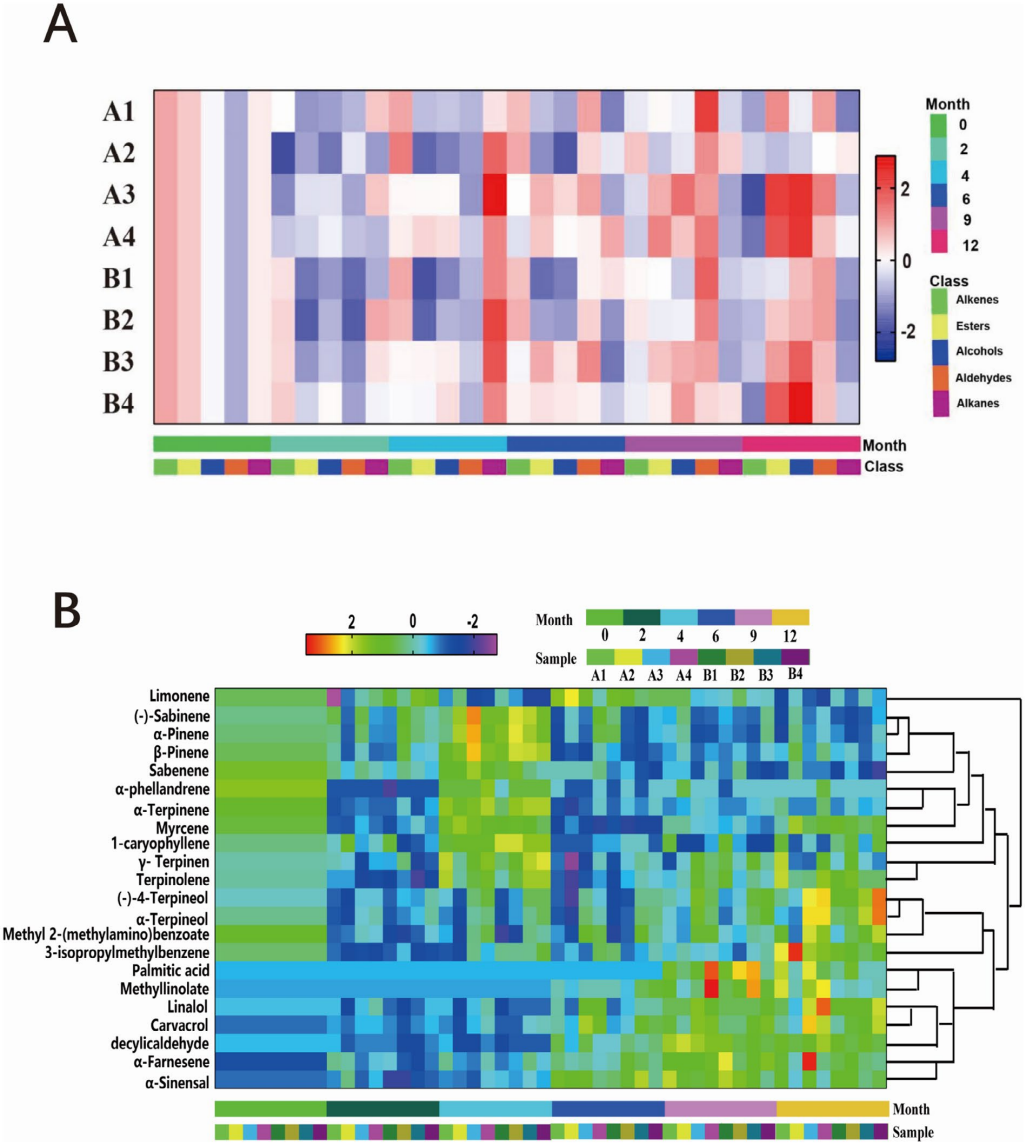
Heat map of relative contents of various volatile components of GCP in different packaging materials.

SPSS 25.0 was used to normalize the total volatile component content of GCP stored under various conditions. The resulting relative content thermogram is shown in Figure 8. After 1 year of storage, alkenes and alkanes generally decreased, while aldehyde levels increased. However, esters, alcohols, and alkanes exhibited varying patterns depending on the storage temperature and container type. Notably, the levels of esters and alcohols in GCP increased significantly after 1 year of storage in glass and iron cans at high temperature. These results suggest that the volatile components in GCP undergo specific changes under different storage conditions. The changes in alkenes, aldehydes, and alkanes followed similar trends, while the patterns for esters and aldehydes varied with different storage conditions.

**FIGURE 9:**
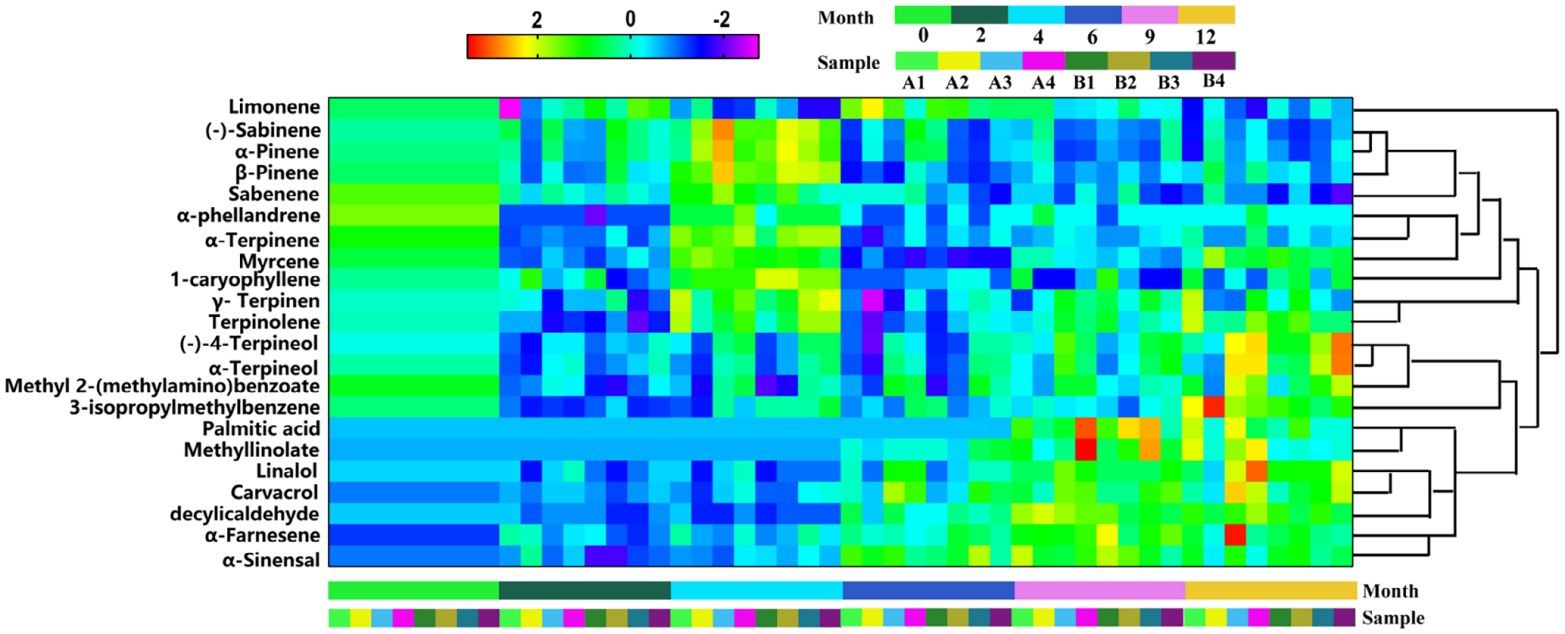
Heat map of relative contents of 22 volatile components of GCP in different packaging materials.

A total of 61 volatile components were identified in the volatile oil stored under different temperatures and in various containers, including 19 alkenes, 11 esters, 15 alcohols, 7 aldehydes, 2 alkanes, and 7 other components. Among these, 22 major volatile components, each with a relative content greater than 0.10%, were selected for further analysis. The relative content data for these 22 components are presented in Table A2 in the Appendix A. SPSS 25.0 was employed to normalize the relative content of the 22 major volatile components, and the resulting thermal spectra are shown in Figure 9.

Of the identified volatile components, 12 were terpenes, including limonene, platylinene, α‐pinene, β‐pinene, sabinene, α‐aquicene, α‐terpinene, myrcene, 1‐caryophyllene, γ‐terpinene, isoterpinene, and α‐farnesene. Notably, limonene and γ‐terpinene exhibited the highest relative contents. After 1 year of storage under varying temperatures and in different containers, the relative content of α‐farnesene increased, while isoterpinene and myrcene showed no significant change. The relative content of most other alkenes decreased, with the contents of specific alkenes, such as 1‐caryophyllene and γ‐terpinene, varying depending on the storage conditions.

Two esters were identified: methyl 2‐(methylamino) benzoate and methyl linoleic acid. The content of methyl 2‐(methylamino) benzoate, a characteristic component of GCP, remained relatively stable in most samples, except for a decrease in the relative content observed in the gunny bag storage. Three alcohols were detected: α‐terpineol, linalool, and (−)‐4‐terpineol. Notably, the relative content of linalool increased, with a more significant rise observed in GCP stored in tin cans. Two aldehydes were also identified, and their relative contents increased after 1 year of storage under various temperature and container conditions. Additionally, the relative contents of three other components, namely, m‐isopropyl toluene, palmitic acid, and carvacrol, also increased after 1 year of storage under different conditions. These findings suggest that the volatile components of GCP undergo continuous transformation during long‐term storage and aging, with the specific effects of temperature and storage containers on the transformation of characteristic volatile components varying accordingly.

## 4 Conclusion

During long‐term storage, temperature, humidity, and storage containers significantly influence the transformation dynamics of key components in GCP. Volatile compounds undergo continuous changes, with storage conditions affecting their transformation patterns. Low temperature and low humidity conditions are unfavorable for the aging process of GCP, while prolonged exposure to high temperature or high humidity accelerates the formation of compounds such as ferulic acid. However, these conditions also lead to a marked reduction in the primary component hesperidin and the characteristic component 2‐(methylamino) benzoate. Such substantial declines may hinder the identification of GCP and Tangerine peel, ultimately compromising the quality evaluation of the medicinal material. Our findings further reveal that GCP stored in PE bags exhibits minimal reductions in total flavonoids and hesperidin, whereas GCP stored in jute bags experiences a pronounced decrease in methyl 2‐(methylamino) benzoate. Additionally, the concentrated aroma of GCP stored in tin cans appears to be associated with an increased relative content of linalool. These results suggest that, while natural aging of GCP has a scientific foundation, mass production requires precise control of storage temperature and humidity to ensure consistent quality.

## Author Contributions

**Mengdie Peng, Jinji Deng** and **Jiepei Xu:** conceptualization, visualization, investigation, and writing – original draft. **Baizhong Chen:** methodology, software, and data curation. **Xiaojing Deng:** methodology, software, and data curation. **Yi Cai:** methodology, software, and data curation. **Wen Liu** and **Guodong Zheng:** methodology, software, data curation, validation, supervision, and writing – review and editing. All authors agree to be accountable for all aspects of the work ensuring integrity and accuracy.

## Ethics Statement

The authors have nothing to report.

## Consent

Written informed consent was obtained from all study participants.

## Conflicts of Interest

The authors declare no conflicts of interest.

## Data Availability

This is an open access article under the terms of the Creative Commons Attribution License, which permits use, distribution, and reproduction in any medium, provided the original work is properly cited.

## Appendix A

**TABLE A1:** Volatile component contents of Guang Chenpi in different storage environments.

No.	RT	Compound	CAS number	ID	Relative contents of various volatile components (%)					
					0 months	2 months	4 months	6 months	9 months	12 months
1	6.33	(‐)‐Sabinene	58037‐87‐9	A	0.62	0.66	0.63	0.47	0.54	0.42
				B	0.62	0.61	0.73	0.66	0.45	0.47
				C	0.62	0.64	0.83	0.47	0.66	0.46
				D	0.62	0.78	0.76	0.64	0.55	0.65
2	6.61	α‐Pinene	7785‐70‐8	A	2.08	2.05	2.05	1.58	1.84	1.50
				B	2.08	2.01	2.51	2.17	1.56	1.66
				C	2.08	2.13	2.73	1.63	2.24	1.73
				D	2.08	2.44	2.49	2.03	1.78	2.13
3	8.35	Sabenene	3387‐41‐5	A	0.21	0.18	0.20	0.17	0.16	0.14
				B	0.21	0.18	0.16	0.16	0.17	0.13
				C	0.21	0.15	0.16	0.14	0.12	0.08
				D	0.21	0.19	0.21	0.20	0.18	0.19
4	8.57	β‐Pinene	127‐91‐3	A	1.56	1.49	1.65	1.25	1.39	1.31
				B	1.56	1.44	1.70	1.43	1.29	1.38
				C	1.56	1.52	1.75	1.19	1.52	1.37
				D	1.56	1.67	1.69	1.53	1.39	1.57
5	9.28	Myrcene	123‐35‐3	A	2.39	2.11	2.49	2.03	2.28	2.35
				B	2.39	2.18	2.38	2.03	2.21	2.28
				C	2.39	2.25	2.50	2.07	2.43	2.36
				D	2.39	2.19	2.47	1.99	2.16	2.38
6	10.17	α‐Phellandrene	99‐83‐2	A	0.12	0.09	0.11	0.10	0.10	0.09
				B	0.12	0.09	0.10	0.10	0.09	0.10
				C	0.12	0.09	0.10	0.07	0.09	0.09
				D	0.12	0.10	0.11	0.10	0.10	0.11
7	10.84	α‐Terpinene	99‐86‐5	A	0.53	1.38	0.56	0.42	0.44	0.44
				B	0.53	0.42	0.52	0.46	0.39	0.46
				C	0.53	0.48	0.51	0.37	0.46	0.44
				D	0.53	0.48	0.52	0.47	0.43	0.47
8	11.33	3‐Isopropylmethylbenzene	535‐77‐3	A	1.44	0.96	0.89	0.99	1.15	1.54
				B	1.44	0.80	1.34	1.03	1.14	1.48
				C	1.44	0.78	1.46	1.36	0.83	1.97
				D	1.44	0.91	1.07	1.33	1.23	1.77
9	11.95	Limonene	138‐86‐3	A	70.58	65.17	68.53	72.29	71.16	70.95
				B	70.58	71.79	69.04	71.44	71.48	68.31
				C	70.58	70.85	69.61	73.49	70.77	69.76
				D	70.58	69.96	69.24	69.71	70.25	68.99
10	13.8	γ‐Terpinen	99‐85‐4	A	17.38	17.30	18.96	16.64	16.76	17.26
				B	17.38	16.77	17.76	16.65	16.42	18.11
				C	17.38	18.17	17.90	16.43	17.51	18.38
				D	17.38	17.71	17.65	17.73	17.23	17.60
11	15.76	Terpinolene	586‐62‐9	A	0.99	0.93	1.14	0.90	0.94	1.00
				B	0.99	0.85	1.00	0.88	0.91	1.09
				C	0.99	0.94	0.99	0.87	0.99	1.09
				D	0.99	0.90	1.00	0.95	0.94	1.02
12	24.52	(‐)‐4‐Terpineol	20126‐76‐5	A	0.23	0.18	0.22	0.18	0.22	0.24
				B	0.23	0.15	0.22	0.19	0.21	0.30
				C	0.23	0.12	0.12	0.07	0.17	0.18
				D	0.23	0.19	0.20	0.21	0.22	0.24
13	26.32	α‐Terpineol	98‐55‐5	A	0.31	0.19	0.22	0.19	0.23	0.27
				B	0.31	0.15	0.23	0.20	0.24	0.37
				C	0.31	0.11	0.10	0.06	0.17	0.20
				D	0.31	0.21	0.22	0.25	0.27	0.30
14	27.74	Decylicaldehyde	112‐31‐2	A	0.05	0.05	0.05	0.09	0.11	0.09
				B	0.05	0.03	0.02	0.07	0.08	0.09
				C	0.05	0.00	0.00	0.03	0.07	0.06
				D	0.05	0.02	0.03	0.06	0.09	0.09
15	31.24	Carvacrol	499‐75‐2	A	0.08	0.10	0.09	0.15	0.19	0.11
				B	0.08	0.06	0.07	0.10	0.19	0.17
				C	0.08	0.04	0.03	0.01	0.02	0.00
				D	0.08	0.08	0.10	0.21	0.22	0.13
16	33.42	Methyl 2‐(methylamino) benzoate	85‐91‐6	A	1.59	1.00	1.14	1.10	1.00	1.00
				B	1.59	0.85	0.83	1.23	1.14	1.63
				C	1.59	0.36	0.16	0.21	0.22	0.14
				D	1.59	1.05	1.23	1.45	1.38	1.21
17	33.66	1‐Caryophyllene	87‐44‐5	A	0.13	0.12	0.14	0.10	0.10	0.09
				B	0.13	0.16	0.19	0.12	0.13	0.16
				C	0.13	0.17	0.19	0.14	0.13	0.16
				D	0.13	0.13	0.16	0.01	0.11	0.11
18	35.07	α‐Farnesene	502‐61‐4	A	0.13	0.31	0.31	0.33	0.34	0.25
				B	0.13	0.27	0.27	0.28	0.41	0.48
				C	0.13	0.25	0.29	0.34	0.51	0.52
				D	0.13	0.21	0.17	0.25	0.37	0.27
19	36.81	Diethyl phthalate	84‐66‐2	A	0.00	0.00	0.00	0.00	0.02	0.02
				B	0.00	0.00	0.00	0.00	0.02	0.03
				C	0.00	0.00	0.00	0.00	0.08	0.11
				D	0.00	0.00	0.00	0.00	0.00	0.01
20	38.33	α‐Sinensal	17909‐77‐2	A	0.21	0.23	0.23	0.47	0.45	0.36
				B	0.21	0.28	0.36	0.32	0.56	0.49
				C	0.21	0.22	0.27	0.53	0.54	0.41
				D	0.21	0.19	0.31	0.35	0.46	0.27
20	40.99	Palmitic acid	57‐10‐3	A	0.00	0.00	0.00	0.00	0.04	0.07
				B	0.00	0.00	0.00	0.00	0.12	0.05
				C	0.00	0.00	0.00	0.00	0.06	0.05
				D	0.00	0.00	0.00	0.00	0.06	0.02

**TABLE A2:** Volatile component contents of Guang Chenpi in different packaging materials.

No.	RT	Compound	CAS number	ID	Relative contents of various volatile components (%)					
					0 month	2 month	4 month	6 month	9 month	12 month
1	6.33	(‐)‐Sabinene	58037‐87‐9	A1	0.62	0.66	0.63	0.47	0.54	0.45
				A2	0.62	0.50	0.77	0.58	0.63	0.61
				A3	0.62	0.66	0.88	0.51	0.49	0.51
				A4	0.62	0.53	0.73	0.69	0.50	0.57
				B1	0.62	0.52	0.73	0.63	0.54	0.49
				B2	0.62	0.69	0.81	0.49	0.51	0.46
				B3	0.62	0.63	0.79	0.46	0.50	0.49
				B4	0.62	0.59	0.73	0.55	0.62	0.54
2	6.61	α‐Pinene	7785‐70‐8	A1	2.08	2.05	2.05	1.58	1.87	1.51
				A2	2.08	1.67	2.52	1.94	2.01	2.07
				A3	2.08	2.11	2.75	1.69	1.62	1.76
				A4	2.08	1.76	2.33	2.17	1.64	1.91
				B1	2.08	1.75	2.42	2.15	1.78	1.76
				B2	2.08	2.21	2.62	1.66	1.71	1.60
				B3	2.08	2.08	2.50	1.60	1.64	1.68
				B4	2.08	1.97	2.35	1.84	2.06	1.93
3	8.35	Sabenene	3387‐41‐5	A1	0.21	0.18	0.20	0.17	0.16	0.14
				A2	0.21	0.16	0.20	0.17	0.16	0.18
				A3	0.21	0.18	0.22	0.17	0.14	0.15
				A4	0.21	0.17	0.20	0.18	0.17	0.15
				B1	0.21	0.16	0.19	0.15	0.15	0.13
				B2	0.21	0.18	0.21	0.16	0.18	0.16
				B3	0.21	0.17	0.18	0.14	0.14	0.13
				B4	0.21	0.16	0.17	0.13	0.13	0.12
4	8.57	β‐Pinene	127‐91‐3	A1	1.56	1.49	1.65	1.25	1.38	1.41
				A2	1.56	1.31	1.70	1.31	1.46	1.58
				A3	1.56	1.46	1.87	1.25	1.33	1.37
				A4	1.56	1.33	1.71	1.48	1.38	1.53
				B1	1.56	1.34	1.69	1.39	1.35	1.41
				B2	1.56	1.59	1.80	1.30	1.42	1.45
				B3	1.56	1.42	1.79	1.27	1.34	1.33
				B4	1.56	1.43	1.76	1.33	1.44	1.42
5	9.28	Myrcene	123‐35‐3	A1	2.39	2.11	2.49	2.03	2.30	2.28
				A2	2.39	2.10	2.55	2.16	2.30	2.56
				A3	2.39	2.20	2.50	2.07	2.23	2.37
				A4	2.39	2.14	2.45	2.00	2.22	2.40
				B1	2.39	2.08	2.43	2.09	2.19	2.47
				B2	2.39	2.17	2.46	2.00	2.25	2.39
				B3	2.39	2.25	2.43	2.02	2.17	2.35
				B4	2.39	2.16	2.36	2.02	2.14	2.39
6	10.17	α‐phellandrene	99‐83‐2	A1	0.12	0.09	0.11	0.10	0.10	0.10
				A2	0.12	0.09	0.11	0.09	0.11	0.10
				A3	0.12	0.09	0.11	0.09	0.10	0.10
				A4	0.12	0.09	0.12	0.10	0.10	0.11
				B1	0.12	0.08	0.10	0.09	0.09	0.10
				B2	0.12	0.09	0.11	0.10	0.10	0.10
				B3	0.12	0.09	0.11	0.09	0.10	0.10
				B4	0.12	0.09	0.11	0.10	0.10	0.10
7	10.84	α‐Terpinene	99‐86‐5	A1	0.53	1.38	0.56	0.42	0.44	0.49
				A2	0.53	0.43	0.54	0.39	0.45	0.44
				A3	0.53	0.44	0.55	0.43	0.47	0.44
				A4	0.53	0.43	0.57	0.47	0.46	0.50
				B1	0.53	0.43	0.50	0.43	0.44	0.44
				B2	0.53	0.47	0.56	0.46	0.44	0.47
				B3	0.53	0.42	0.57	0.44	0.46	0.45
				B4	0.53	0.45	0.57	0.47	0.47	0.46
8	11.33	3‐isopropylmethylbenzene	535‐77‐3	A1	1.44	0.96	0.89	0.99	1.14	2.11
				A2	1.44	0.78	0.78	1.14	1.30	2.51
				A3	1.44	0.84	1.38	0.96	1.17	1.94
				A4	1.44	0.79	1.11	1.50	1.21	1.84
				B1	1.44	0.91	1.37	1.45	1.12	1.75
				B2	1.44	1.14	1.36	0.97	0.91	1.64
				B3	1.44	0.80	1.37	1.08	1.22	1.47
				B4	1.44	0.84	1.56	1.28	1.32	1.71
9	11.95	Limonene	138‐86‐3	A1	70.58	65.17	68.53	72.29	70.60	67.23
				A2	70.58	68.31	70.27	73.52	70.60	69.42
				A3	70.58	69.70	67.48	71.93	69.02	68.00
				A4	70.58	70.26	67.68	69.61	69.13	66.93
				B1	70.58	71.40	69.76	71.84	69.38	69.50
				B2	70.58	69.99	68.61	71.72	70.53	68.16
				B3	70.58	72.07	66.95	70.35	69.31	69.64
				B4	70.58	71.69	66.94	70.62	69.45	68.72
10	13.8	γ‐Terpinen	99‐85‐4	A1	17.38	17.30	18.96	16.64	16.32	18.95
				A2	17.38	17.13	17.46	15.27	17.17	16.66
				A3	17.38	16.15	18.24	16.01	18.07	16.56
				A4	17.38	16.89	18.61	17.27	17.65	18.13
				B1	17.38	16.87	17.68	16.36	17.88	17.16
				B2	17.38	17.65	18.05	17.11	17.23	18.16
				B3	17.38	15.97	18.91	17.48	18.02	17.15
				B4	17.38	16.51	19.12	17.24	17.42	16.73
11	15.76	Terpinolene	586‐62‐9	A1	0.99	0.93	1.14	0.90	0.95	1.15
				A2	0.99	0.93	0.99	0.82	0.98	1.00
				A3	0.99	0.85	1.04	0.89	1.05	1.01
				A4	0.99	0.88	1.09	0.93	1.02	1.12
				B1	0.99	0.86	0.98	0.87	1.05	1.06
				B2	0.99	0.92	1.04	0.94	0.95	1.10
				B3	0.99	0.82	1.13	0.98	1.00	1.02
				B4	0.99	0.87	1.13	0.96	0.97	1.02
12	17.33	Linalool	78‐70‐6	A1	0.05	0.05	0.05	0.06	0.07	0.07
				A2	0.05	0.03	0.03	0.04	0.07	0.05
				A3	0.05	0.05	0.05	0.08	0.09	0.10
				A4	0.05	0.06	0.06	0.08	0.08	0.12
				B1	0.05	0.04	0.03	0.04	0.07	0.08
				B2	0.05	0.03	0.04	0.05	0.07	0.08
				B3	0.05	0.04	0.04	0.06	0.07	0.08
				B4	0.05	0.05	0.04	0.06	0.08	0.10
13	24.52	(‐)‐4‐Terpineol	20126‐76‐5	A1	0.23	0.18	0.22	0.18	0.24	0.25
				A2	0.23	0.15	0.17	0.12	0.22	0.22
				A3	0.23	0.22	0.26	0.25	0.32	0.37
				A4	0.23	0.22	0.28	0.22	0.27	0.38
				B1	0.23	0.18	0.17	0.16	0.20	0.29
				B2	0.23	0.21	0.20	0.17	0.22	0.29
				B3	0.23	0.20	0.27	0.25	0.28	0.34
				B4	0.23	0.24	0.27	0.25	0.29	0.41
14	26.32	α‐Terpineol	98‐55‐5	A1	0.31	0.19	0.22	0.19	0.27	0.25
				A2	0.31	0.16	0.17	0.13	0.23	0.22
				A3	0.31	0.27	0.30	0.33	0.41	0.51
				A4	0.31	0.29	0.32	0.29	0.33	0.51
				B1	0.31	0.19	0.18	0.17	0.21	0.33
				B2	0.31	0.22	0.23	0.20	0.27	0.35
				B3	0.31	0.25	0.31	0.33	0.34	0.47
				B4	0.31	0.30	0.34	0.32	0.38	0.56
15	27.74	Decylicaldehyde	112‐31‐2	A1	0.05	0.05	0.05	0.09	0.13	0.11
				A2	0.05	0.03	0.02	0.05	0.14	0.09
				A3	0.05	0.04	0.02	0.09	0.13	0.11
				A4	0.05	0.04	0.04	0.06	0.12	0.13
				B1	0.05	0.04	0.02	0.06	0.12	0.11
				B2	0.05	0.02	0.03	0.08	0.09	0.09
				B3	0.05	0.02	0.03	0.07	0.10	0.11
				B4	0.05	0.04	0.03	0.08	0.09	0.09
16	31.24	Carvacrol	499‐75‐2	A1	0.08	0.10	0.09	0.15	0.20	0.16
				A2	0.08	0.08	0.05	0.11	0.15	0.12
				A3	0.08	0.11	0.11	0.28	0.26	0.33
				A4	0.08	0.11	0.14	0.24	0.25	0.29
				B1	0.08	0.09	0.07	0.10	0.17	0.15
				B2	0.08	0.06	0.07	0.12	0.17	0.20
				B3	0.08	0.09	0.13	0.23	0.23	0.22
				B4	0.08	0.11	0.14	0.19	0.25	0.29
17	33.42	Methyl 2‐(methylamino)benzoate	85‐91‐6	A1	1.59	1.00	1.14	1.10	1.24	1.58
				A2	1.59	1.05	0.81	0.93	1.10	1.05
				A3	1.59	1.28	1.40	1.60	1.57	1.99
				A4	1.59	1.23	1.53	1.52	1.57	1.83
				B1	1.59	0.87	0.71	0.77	1.24	1.20
				B2	1.59	0.78	0.80	1.00	1.17	1.49
				B3	1.59	1.00	1.41	1.60	1.36	1.68
				B4	1.59	1.23	1.34	1.37	1.31	1.94
18	33.66	1‐caryophyllene	87‐44‐5	A1	0.13	0.12	0.14	0.10	0.14	0.14
				A2	0.13	0.15	0.15	0.10	0.09	0.10
				A3	0.13	0.11	0.15	0.10	0.09	0.12
				A4	0.13	0.12	0.15	0.11	0.11	0.10
				B1	0.13	0.14	0.17	0.11	0.15	0.13
				B2	0.13	0.09	0.17	0.12	0.11	0.14
				B3	0.13	0.10	0.16	0.12	0.09	0.11
				B4	0.13	0.11	0.16	0.10	0.09	0.14
19	35.07	α‐Farnesene	502‐61‐4	A1	0.13	0.31	0.31	0.33	0.40	0.38
				A2	0.13	0.29	0.20	0.28	0.41	0.27
				A3	0.13	0.18	0.18	0.30	0.40	0.71
				A4	0.13	0.24	0.28	0.26	0.44	0.32
				B1	0.13	0.25	0.18	0.28	0.56	0.36
				B2	0.13	0.15	0.21	0.38	0.34	0.40
				B3	0.13	0.12	0.32	0.35	0.42	0.33
				B4	0.13	0.18	0.24	0.31	0.47	0.31
20	38.33	α‐Sinensal	17909‐77‐2	A1	0.21	0.23	0.23	0.47	0.55	0.42
				A2	0.21	0.35	0.22	0.42	0.41	0.28
				A3	0.21	0.20	0.19	0.46	0.40	0.45
				A4	0.21	0.26	0.28	0.35	0.46	0.30
				B1	0.21	0.10	0.27	0.37	0.54	0.42
				B2	0.21	0.10	0.24	0.41	0.49	0.44
				B3	0.21	0.18	0.29	0.54	0.42	0.35
				B4	0.21	0.20	0.25	0.34	0.34	0.39
21	40.9	Palmitic acid	57‐10‐3	A1	0.00	0.00	0.00	0.00	0.08	0.11
				A2	0.00	0.00	0.00	0.00	0.04	0.02
				A3	0.00	0.00	0.00	0.00	0.06	0.12
				A4	0.00	0.00	0.00	0.00	0.16	0.05
				B1	0.00	0.00	0.00	0.00	0.08	0.03
				B2	0.00	0.00	0.00	0.00	0.13	0.08
				B3	0.00	0.00	0.00	0.00	0.14	0.02
				B4	0.00	0.00	0.00	0.00	0.03	0.02
22	41.48	Methyl Linolate	112‐63‐0	A1	0.00	0.00	0.00	0.03	0.08	0.14
				A2	0.00	0.00	0.00	0.01	0.02	0.03
				A3	0.00	0.00	0.00	0.03	0.05	0.12
				A4	0.00	0.00	0.00	0.03	0.21	0.15
				B1	0.00	0.00	0.00	0.03	0.06	0.02
				B2	0.00	0.00	0.00	0.01	0.09	0.03
				B3	0.00	0.00	0.00	0.06	0.17	0.02
				B4	0.00	0.00	0.00	0.07	0.07	0.03
